# From Gut to Fat: Intestinal Epithelial Exosomes Target PDGFRα ^+^ Progenitors to Promote Lipogenesis and Counteract Subcutaneous Adipose Tissue Atrophy in Aging

**DOI:** 10.1111/acel.70625

**Published:** 2026-07-12

**Authors:** Tingting Huang, Ye Huang, Yin Zhou, Xuanbei Lu, Lijun Yang, Jing Yu, Yunlu Sheng, Fan Xia, Guoxian Ding, Yifan Lv, Shan Lv

**Affiliations:** ^1^ Division of Geriatric Endocrinology The First Affiliated Hospital of Nanjing Medical University Nanjing China; ^2^ Department of Geriatrics, the First Affiliated Hospital, School of Medicine Zhejiang University Hangzhou Zhejiang China; ^3^ Division of Geriatric Liyang People's Hospital, Liyang Branch Hospital of Jiangsu Province Hospital Changzhou China

**Keywords:** aging, exosome, lipid droplet, small intestinal epithelium, white adipose tissue

## Abstract

Age‐related subcutaneous adipose tissue (SAT) atrophy is a hallmark of aging, contributing to metabolic dysfunction and systemic aging. The mechanisms underlying SAT atrophy and potential therapeutic strategies remain poorly understood. Here, we report that small intestinal epithelium‐derived exosomes (SI‐Exos) mediate gut‐adipose communication and play a pivotal role in age‐related SAT remodeling. We found that the miRNA cargo of SI‐Exos undergoes significant age‐related changes. Administration of young SI‐Exos to aged mice enhanced lipid droplet formation, reversed SAT atrophy, and reduced inflammation in visceral adipose tissue (VAT). These beneficial effects were mediated by young SI‐Exos targeting PDGFRα^+^ progenitor cells, the major adipocyte precursors in SAT. Mechanistically, young SI‐Exos were enriched with miR‐379‐5p, which targeted Usp34, a negative regulator of lipogenesis. Inhibition of Usp34 downregulated the Wnt/β‐catenin pathway, promoting lipid droplet formation and differentiation of PDGFRα^+^ progenitor cells. Single‐cell RNA sequencing analysis further confirmed that young SI‐Exos enhanced lipid transport and synthesis in SAT cell populations. Additionally, NK cells were increased. Our findings reveal a previously unrecognized role of SI‐Exos in regulating SAT progenitor cell dynamics through the miR‐379‐5p/Usp34/Wnt/β‐catenin axis, offering a potential therapeutic strategy for combating age‐associated SAT atrophy and promoting healthy aging.

## Graphical abstract

Young small intestinal epithelial‐derived exosomes (young‐SI‐Exos) deliver miR‐379‐5p, which targets Usp34 to suppress the Wnt/β‐catenin pathway, thereby promoting adipocyte differentiation, enhancing lipid metabolism, and increasing lipid droplets in PDGFRα^+^ progenitors, ultimately reversing age‐related subcutaneous adipose tissue (SAT) atrophy.

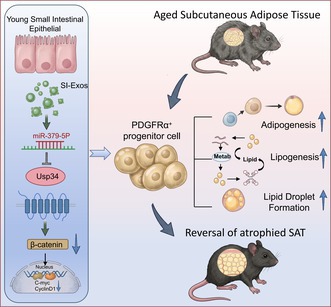

## 1 Introduction

Adipose tissue, the most widely distributed metabolic organ in the body, is a key regulator of organismal aging (Ou et al. 2022). Multi‐organ transcriptomic profiling has confirmed that adipose tissue exhibits significant aging‐associated gene expression changes as early as 12 months of age in mice (equivalent to approximately 36 years in humans), preceding similar changes in other organs (Wu et al. 2023). In addition, adipose tissue signaling has been identified as a promising therapeutic target for anti‐aging interventions (Miller et al. 2017; Song et al. 2023). Therefore, adipose tissue aging may serve as an early driver of systemic aging and may have profound consequences for organ function. Previous studies have primarily focused on visceral adipose tissue (VAT) accumulation and function, whereas the aging and atrophy of subcutaneous adipose tissue (SAT) have received less attention (Wang, Song, et al. 2025).

Studies have shown that adipose depots at different anatomical locations differ in composition and function (Zwick et al. 2018). SAT is the largest fat storage depot and the primary site for storing excess energy as triglycerides, accounting for approximately 80% of total body fat (Neamat‐Allah et al. 2014; Reddy et al. 2019). Numerous studies have demonstrated the metabolic benefits of SAT. Animal transplantation studies have shown that mice receiving SAT grafts exhibit significant improvements in body weight, total fat mass, and insulin sensitivity. In contrast, mice receiving VAT grafts do not show these beneficial effects (Zwick et al. 2018). With advancing age, SAT undergoes atrophy, and its capacity to buffer excess lipids declines. Consequently, adipose tissue aging promotes ectopic lipid deposition, chronic low‐grade inflammation, and metabolic dysfunction, which severely impair the function of organs such as the muscle, liver, and heart (Bailin et al. 2022). However, little is known about the mechanisms underlying SAT atrophy or the development of effective therapeutic interventions.

The intestine, a critical organ for nutrient absorption, is closely associated with adipose tissue storage and redistribution. Moschandrea et al. demonstrated that mice with intestine‐specific knockout of DARS2 (encoding aspartyl‐tRNA synthetase 2) exhibited impaired lipid transport and reduced white adipose tissue mass in abdominal and inguinal depots (Moschandrea et al. 2024). Furthermore, Bäckhed et al. showed that suppression of intestinal fasting‐induced adipocyte factor (Fiaf) abolished the gut microbiota‐mediated promotion of fat storage and redistribution (Bäckhed et al. 2004). Notably, previous studies have demonstrated that the function of the gut microbiota depends critically on the microenvironment provided by the intestinal epithelium (Wilmanski et al. 2022). As an important endocrine organ involved in interorgan communication, intestinal epithelial cells secrete exosomes that facilitate interorgan signaling (Krylova and Feng 2023). A recent study published in Cell demonstrated that exosomes, which act as key mediators of cellular function, are nanoscale vesicles with substantial potential for systemic rejuvenation and age reversal (Lei et al. 2025). Our group previously developed a novel method for isolating intestinal epithelial cells from the lamina propria, enabling the specific extraction of small intestinal epithelial exosomes (SI‐Exos) (Xia et al. 2019). In addition, we demonstrated that senescent exosomes accelerate cardiac aging and exacerbate cardiac inflammation and fibrosis (He et al. 2025).

Here, we demonstrated that miR‐379‐5p enriched in young SI‐Exos directly targets Usp34 to regulate lipid droplet formation in PDGFRα^+^ progenitor cells, which represent 60.5% of primary SAT cells. These findings suggest that modulation of SI‐Exos‐mediated adipose tissue dynamics may provide a promising strategy for reversing SAT atrophy. Our study provides mechanistic insights into the prevention of adipose dysfunction and the alleviation of age‐related diseases, thereby promoting healthy aging.

## 2 Results

### 2.1 Young SI‐Exos Promoted Adipogenesis During Aging Independently of Gut Microbiota

Extracellular vesicles mediate intercellular communication by transporting bioactive molecules, and their roles in age‐related diseases have increasingly attracted attention (Krylova and Feng 2023).

**FIGURE 1:**
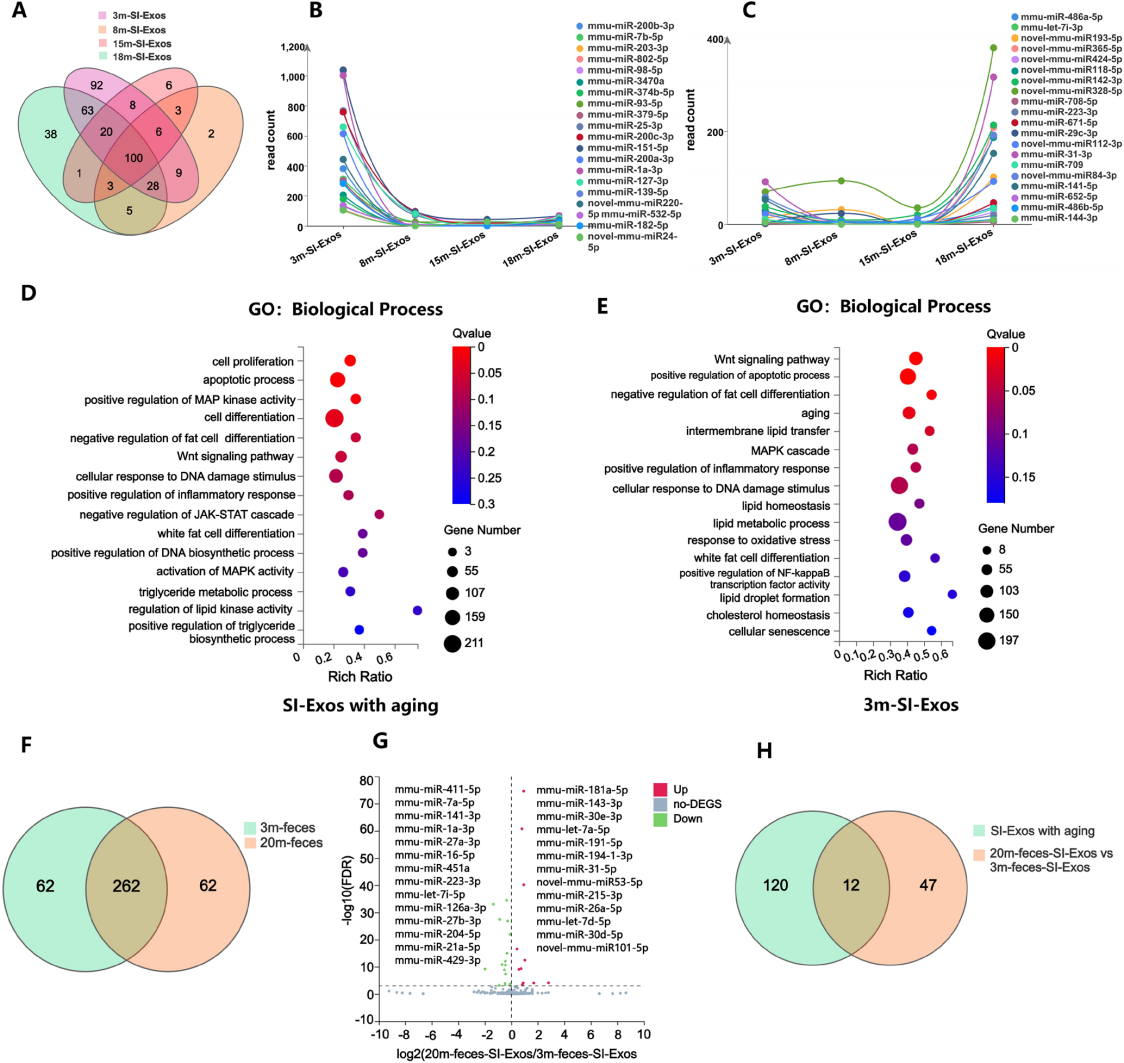
MiRNA high‐throughput sequencing of SI‐Exos in mice of different ages and fecal transplantation mice (A–E) MiRNA high‐throughput sequencing was performed on SI‐Exos extracted from mice aged 3, 8, 15, and 18 months. (A) Venn diagram of differentially expressed miRNAs in SI‐Exos from 3‐, 8‐, 15‐, and 18‐month‐old mice. (B) Line graph showing the top 20 miRNAs with decreasing expression during aging. (C) Line graph showing the top 20 miRNAs with increasing expression during aging. (D) GO enrichment analysis of age‐altered miRNAs. (E) GO enrichment analysis of 3m‐SI‐Exos. (F‐G) FMT experiment: Fecal microbiota from young (3‐month‐old) and aged (20‐month‐old) donors were transplanted into 8‐week‐old germ‐free recipient mice (*n* = 3). Two weeks post‐transplantation, SI‐Exos were extracted for miRNA sequencing. (F) Venn diagram of differentially expressed miRNAs from SI‐Exos in the 3m‐feces and 20m‐feces groups. (G) Volcano plot of differentially expressed miRNAs between 3m‐feces‐SI‐Exos and 20m‐feces‐SI‐Exos. (H) Venn diagram showing miRNAs consistently regulated in both comparisons. SI‐Exos, small intestinal epithelial exosomes; 3m‐/8m‐/15m‐/18m‐SI‐Exos, SI‐Exos from 3‐/8‐/15‐/18‐month‐old mice; 3m‐feces‐SI‐Exos/20m‐feces‐SI‐Exos, SI‐Exos from germ‐free mice transplanted with feces from 3‐/20‐month‐old donors; SI‐Exos with aging, small intestinal epithelial exosomes exhibiting age‐dependent variations (from mice aged 3, 8, 15, and 18 months).

Building on our previous extraction and characterization of SI‐ and serum‐derived exosomes (He et al. 2025; Xia et al. 2019), we performed comprehensive characterization of SI‐Exos in this study (Figure S1A–C). MiRNA sequencing of SI‐Exos from male mice aged 3, 8, 15, and 18 months revealed age‐dependent alterations: 102 miRNAs decreased, and 30 increased with age (Figure 1A, GSE283891 dataset). The top 20 upregulated and top 20 downregulated miRNAs are presented in Figure 1B,C, and the full list is available in Table S2. The predicted target genes of these differentially expressed miRNAs were enriched in pathways including apoptosis, inflammation, DNA damage, white adipocyte differentiation, lipid metabolism, and adipogenesis‐related MAPK and Wnt signaling (Figure 1D). Notably, miRNA target genes from 3m‐SI‐Exos specifically enriched lipid droplet formation, lipid metabolism, and related signaling pathways (Figure 1E).

To determine whether these age‐related changes depend on the gut microbiota, fecal microbiota transplantation (FMT) experiments were performed. Gut microbiota from young (3‐month‐old) and aged (20‐month‐old) donor mice were transferred into 8‐week‐old germ‐free recipient mice. MiRNA sequencing of SI‐Exos from recipient mice identified 262 overlapping miRNAs between the two groups, with 62 unique miRNAs in each group (Figure 1F). Differential analysis revealed 13 upregulated and 14 downregulated miRNAs in the 20m‐feces group compared to the 3m‐feces group (Figure 1G). Integrated analysis of the differentially expressed miRNAs in SI‐Exos from the FMT model (20m‐feces‐SI‐Exos vs. 3m‐feces‐SI‐Exos) and aging‐related SI‐Exos (from mice aged 3, 8, 15, and 18 months) identified only 2 consistently upregulated and 8 consistently downregulated miRNAs (Figure 1H). Furthermore, FMT did not result in significant differences in body weight, fat content, adipose tissue morphology, or adipocyte size (Figure S2A–C). These findings suggested that age‐related SI‐Exos changes occurred independently of the gut microbiota.

### 2.2 Young SI‐Exos Reversed Subcutaneous Fat Atrophy in Aged Mice

**FIGURE 2:**
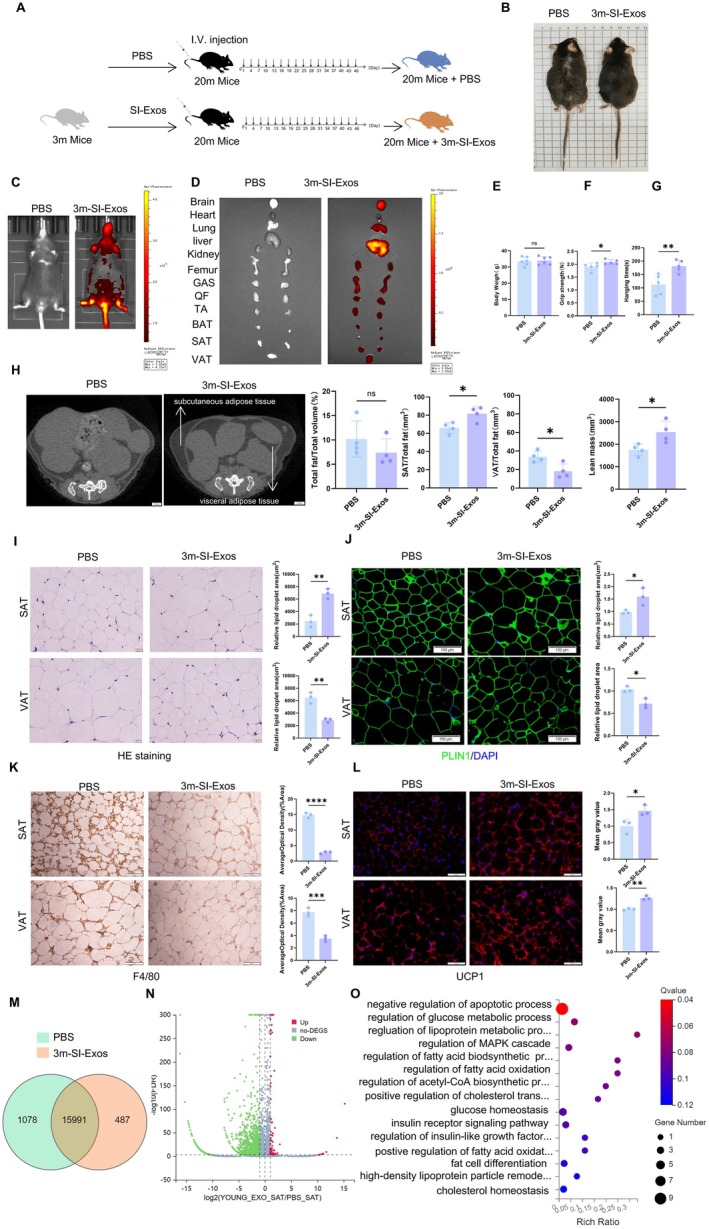
Young SI‐Exos reversed subcutaneous fat atrophy in aged mice 20‐month‐old mice received tail vein injections of SI‐Exos derived from 3‐month‐old mice (100 μg per injection, every 3 days for 2 months, total 20 injections). Control mice received an equal volume of PBS (*n* = 6). (A) Flowchart of the experimental design. (B) Representative images of mice after 20 injections. (C, D) Uptake of PBS and PKH26‐labeled 3m‐SI‐Exos in tissues of wild‐type mice 24 h after administration (*n* = 1). (C) Representative in vivo images demonstrating systemic uptake of PKH26. (D) Representative images of PKH26 uptake in various organs. (E) Body weight (*n* = 6). (F) Grip strength (*n* = 6). (G) Hanging time (*n* = 6). (H) Micro‐CT analysis (*n* = 4). (I) H&E staining of adipocytes in SAT and VAT (*n* = 3). Scale bar, 50 μm. (J) PLIN1 (green, lipid droplet marker) and DAPI (blue, nuclei) immunofluorescence in SAT and VAT (*n* = 3). Scale bar, 100 μm. (K) Immunohistochemistry staining of F4/80 (*n* = 3). Scale bar, 50 μm. (L) UCP1 (red) and DAPI (blue) immunofluorescence (*n* = 3). Scale bar, 50 μm. (M‐O) RNA sequencing analysis of SAT. (M) Venn diagrams of DEGs. (N) Volcano plot of DEGs. (O) GO analysis of enriched pathways. Error bars represent ± SD. Comparisons between groups were analyzed by Student's *t*‐test. *p < 0.05, **p < 0.01, ***p < 0.001. 3m‐SI‐Exos, small intestinal epithelial exosomes from 3‐month‐old mice; DEGs, differentially expressed genes; SAT, subcutaneous adipose tissue; VAT, visceral adipose tissue.

SI‐Exos were extracted from young (3‐month‐old) mice and injected into aged (20‐month‐old) mice to evaluate their effect on aged SAT (Figure 2A). SAT showed uptake of exosomes (Figure 2C,D). The 3m‐SI‐Exos group exhibited a less pronounced aging phenotype, characterized by improved hair condition, significantly increased grip strength, and prolonged hanging times (Figure 2B,E–G). Micro‐CT data indicated that the 3m‐SI‐Exos group showed a significant increase in SAT content and lean mass, along with a trend toward decreased VAT content, although overall body fat percentage remained unchanged (Figure 2H).

HE staining demonstrated an increase in average adipocyte area in SAT and a decrease in VAT in the 3m‐SI‐Exos group (Figure 2I). Perilipin 1 (PLIN1) immunofluorescence staining revealed increased cross‐sectional areas of lipid droplets in SAT and decreased areas in VAT (Figure 2J). F4/80 staining intensity decreased in both SAT and VAT of mice treated with 3m‐SI‐Exos, indicating reduced inflammation. UCP1 intensity increased, consistent with enhanced beiging (Figure 2K,L). Furthermore, expression levels of genes related to adipocyte differentiation, triglyceride and fatty acid synthesis, lipid droplet formation, and lipolysis were significantly higher in the 3m‐SI‐Exos group (Figure S3A).

SAT from both groups was further analyzed using RNA sequencing. Differential expression analysis identified 83 upregulated and 1184 downregulated differentially expressed genes (DEGs) in the 3m‐SI‐Exos group (Figure 2M,N). GO analysis indicated that DEGs primarily enriched in pathways related to lipid synthesis, lipid transport, and apoptosis (Figure 2O).

These results demonstrated that treatment with 3m‐SI‐Exos reversed SAT atrophy, attenuated VAT accumulation, reduced inflammation in SAT and VAT, and promoted beiging.

### 2.3 Young SI‐Exos Promoted Lipid Droplet Formation in PDGFRα ^+^ Progenitor Cells of SAT

**FIGURE 3:**
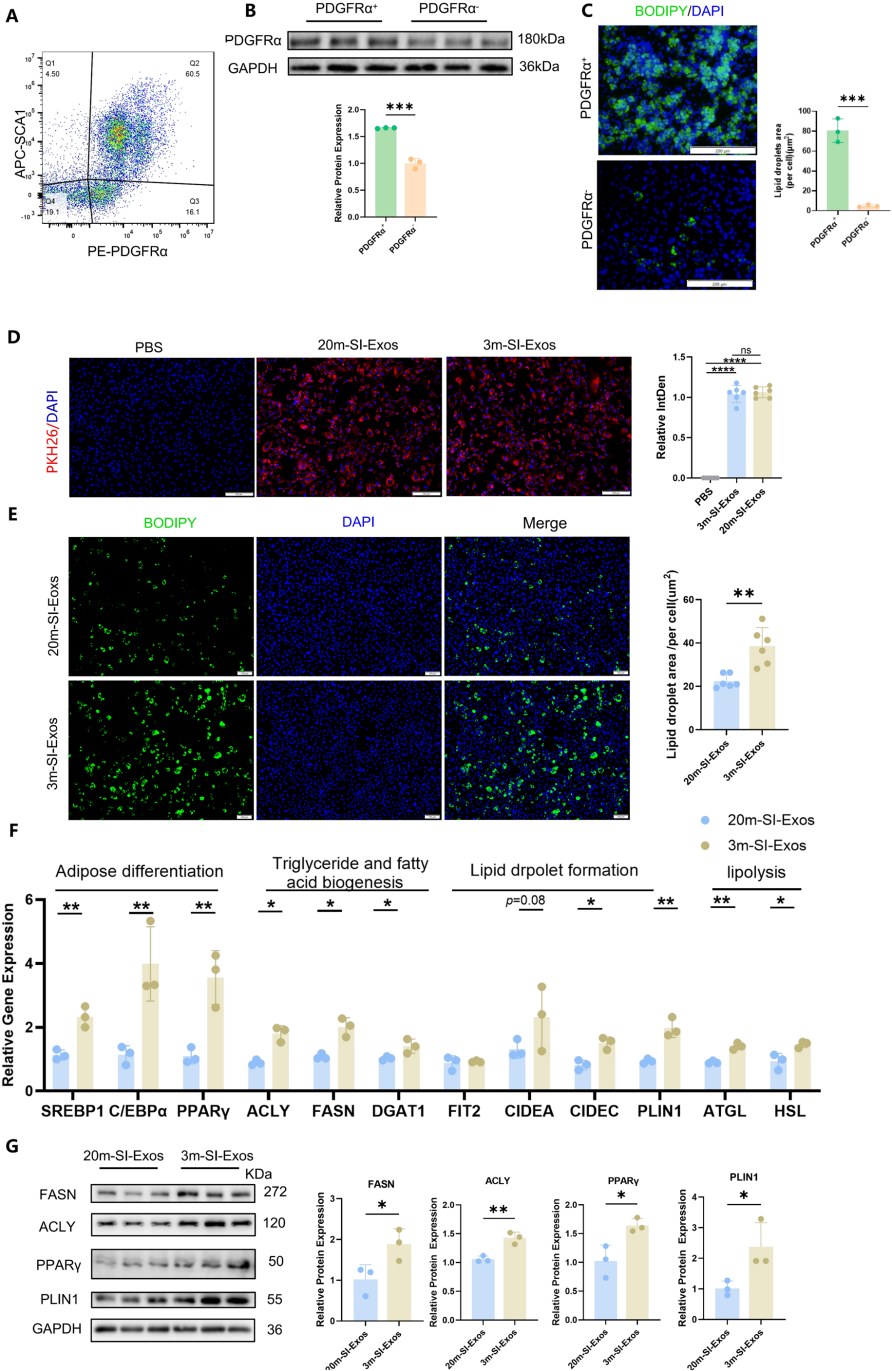
Young SI‐Exos promoted adipogenesis in PDGFRα^+^ progenitor cells of SAT (A) Representative flow cytometry plots showing the gating strategy for PDGFRα^+^ progenitor cells sorted from SAT (*n* = 1). (B‐C) PDGFRα^+^ and PDGFRα^−^ progenitor cells were sorted by magnetic beads and induced to undergo adipogenesis for 6 days. (B) Expression levels of PDGFRα protein (*n* = 3). (C) BODIPY staining of lipid droplets (green) and DAPI staining of nuclei (blue). Scale bar, 200 μm (*n* = 3). (D) Magnetic isolation of PDGFRα^+^ progenitor cells followed by treatment with PKH26 (red)‐labeled young (3m) or aged (20m) SI‐Exos or PBS for 24 h; nuclei stained with DAPI (blue). Scale bar, 200 μm (*n* = 3). (E‐G) PDGFRα^+^ progenitor cells isolated by magnetic beads were co‐cultured with young (3m) or aged (20m) SI‐Exos for 6 days, followed by adipogenic induction. (E) BODIPY staining of lipid droplets (green) and nuclei (blue). Scale bar, 200 μm (*n* = 3). (F) Relative gene expression levels of SREBP1, C/EBPα, PPARγ, FASN, ACLY, DGAT1, ATGL, HSL, FIT2, CIDEA, CIDEC, and PLIN1 (*n* = 3). (G) Relative protein expression levels of FASN, ACLY, PPARγ, and PLIN1 (*n* = 3). Error bars represent ± SD. Comparisons between groups were analyzed by Student's *t*‐test. *p < 0.05, **p < 0.01, ***p < 0.001, ****p < 0.0001. SAT, subcutaneous adipose tissue; SVF, stromal vascular fraction; Young SI‐Exos, small intestinal epithelial exosomes from young mice.

Recent studies have indicated that PDGFRα^+^ progenitor cells are the primary adipocyte progenitor cells (APCs) in WAT and contribute to adipocyte formation. To verify the specific effects of young SI‐Exos on PDGFRα^+^ progenitor cells, the stromal vascular fraction (SVF) from SAT of 3‐week‐old mice was isolated and cultured. Flow cytometry revealed that PDGFRα^+^ progenitor cells accounted for 60.5% of primary SAT cells. PDGFRα^+^ progenitors expressed SCA1, whereas PDGFRα^−^ progenitors did not (Figure 3A). Next, primary SAT cells were sorted using magnetic beads. PDGFRα protein expression was significantly higher in PDGFRα^+^ progenitors, confirming successful sorting (Figure 3B). Bodipy staining showed significantly more lipid droplets in PDGFRα^+^ progenitor cells compared to PDGFRα^−^ cells (Figure 3C).

In addition, PDGFRα^+^ progenitor cells showed higher expression levels of adipogenic genes, including white adipocyte markers, brown adipocyte markers, and adipokine genes (Figure S3B). Single‐cell RNA sequencing (scRNA‐seq) of SAT isolated from both groups further showed predominant PDGFRα expression in preadipocytes, with a marked increase in the proportion of PDGFRα^+^ progenitor cells in the 3m‐SI‐Exos group (Figure S4A–D). Differential expression analysis identified 925 upregulated DEGs and 8 downregulated DEGs in PDGFRα^+^ progenitor cells from the 3m‐SI‐Exos group (Figure S4E). GO analysis indicated enrichment in pathways related to lipid synthesis, lipid metabolism, lipid storage, inflammation, and apoptosis (Figure S4F). These results suggested that PDGFRα^+^ progenitor cells mediated the effects of 3m‐SI‐Exos. Moreover, PDGFRα^+^ progenitor cells exhibited bipotent progenitor potential for white and beige adipogenesis.

PDGFRα^+^ progenitor cells internalized exosomes, and uptake rates for exosomes derived from mice of different ages showed no significant differences (Figure 3D). However, the total lipid droplet area per nucleus was significantly greater in PDGFRα^+^ progenitor cells treated with 3m‐SI‐Exos compared to those treated with 20m‐SI‐Exos (Figure 3E). Furthermore, expression levels of genes involved in adipocyte differentiation, triglyceride and fatty acid synthesis, lipid droplet formation, and lipolysis were significantly higher in the 3m‐SI‐Exos group (Figure 3F). Expression levels of lipid droplet‐associated proteins also increased (Figure 3G). Collectively, these data indicated that young SI‐Exos promoted lipid droplet accumulation in PDGFRα^+^ progenitor cells by enhancing adipocyte differentiation, lipid metabolism, and lipid droplet formation and fusion.

### 2.4 MiR‐379‐5p Promoted Lipid Droplet Accumulation in PDGFRα ^+^ Progenitor Cells of SAT by Targeting Usp34

**FIGURE 4:**
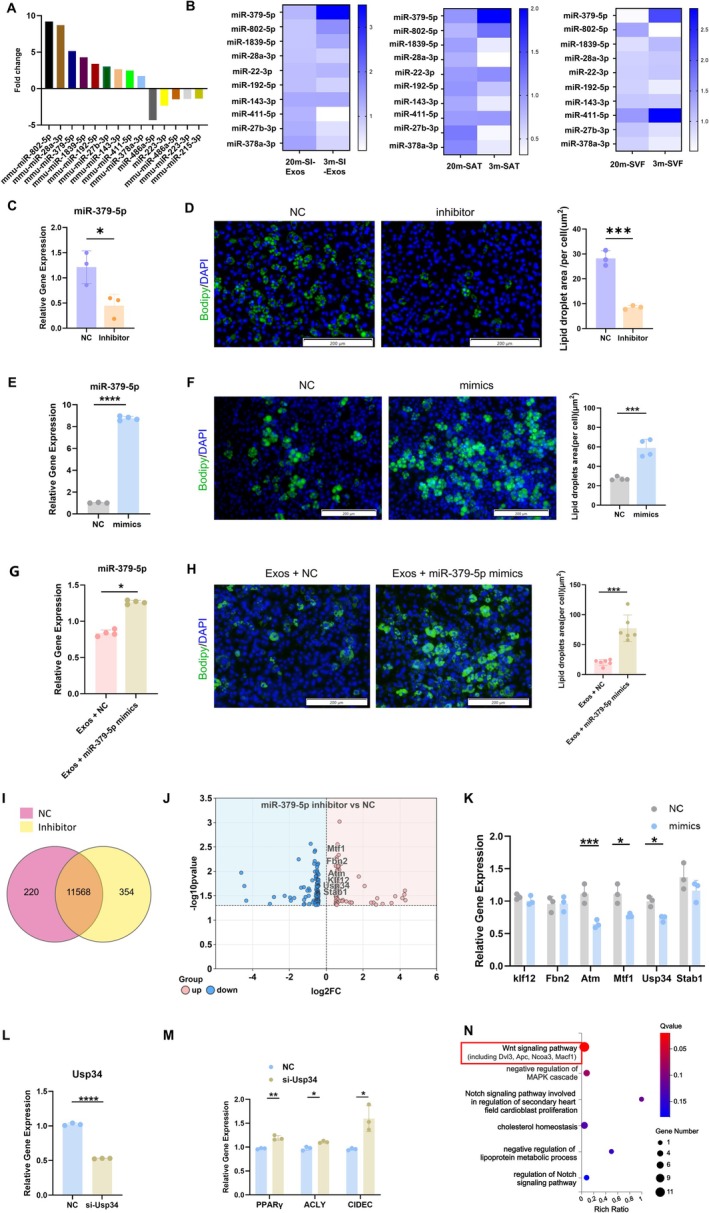
Screening of target miRNAs and target genes (A) MiRNAs consistently upregulated and downregulated in SI‐Exos and serum exosomes comparing young (3‐month‐old) to aged (18‐month‐old) mice. Fold‐change represents young versus aged (young/aged) ratios derived from SI‐Exos. (B) RT‐qPCR analysis of miRNA expression in SAT, SVF, and SI‐Exos from young (3‐month‐old) and aged (20‐month‐old) mice (*n* = 3). (C‐D) PDGFRα^+^ progenitor cells from SAT were isolated and transfected with miR‐379‐5p inhibitor or NC. (C) RT‐qPCR validation of miR‐379‐5p inhibition at 48 h (*n* = 3). (D) Cells underwent adipogenic differentiation for 6 days, followed by BODIPY (green, lipid droplets) and DAPI (blue, nuclei) staining. Scale bar, 200 μm (*n* = 3). (E, F) PDGFRα^+^ progenitor cells from SAT were transfected with miR‐379‐5p mimics or NC. (E) RT‐qPCR validation of miR‐379‐5p overexpression at 48 h (NC, *n* = 3; mimics, *n* = 4). (F) Cells underwent adipogenic differentiation for 6 days, followed by BODIPY (green, lipid droplets) and DAPI (blue, nuclei) staining. Scale bar, 200 μm (*n* = 4). (G, H) SI‐Exos from aged (20‐month‐old) mice were transfected with miR‐379‐5p mimics or NC, followed by co‐culture with PDGFRα^+^ progenitor cells. (G) RT‐qPCR validation of miR‐379‐5p overexpression at 48 h (*n* = 4). (H) Cells underwent adipogenic differentiation for 6 days, followed by BODIPY (green, lipid droplets) and DAPI (blue, nuclei) staining. Scale bar, 200 μm (*n* = 6). (I–J, N) PDGFRα^+^ progenitor cells were transfected with miR‐379‐5p inhibitor or NC, followed by RNA‐seq at 48 h (*n* = 3). (I) Venn diagram of DEGs. (J) Volcano plot of top 100 upregulated (pink) and downregulated (blue) DEGs. (K) RT‐qPCR validation of six target genes after miR‐379‐5p overexpression (*n* = 3). (L, M) PDGFRα^+^ progenitor cells transfected with siRNAs targeting Usp34 or NC. (L) Knockdown efficiency of Usp34 (*n* = 3). (M) Relative gene expression of PPARγ, ACLY, and CIDEC (*n* = 3). (N) GO enrichment analysis of pathways enriched by upregulated DEGs in the inhibitor group versus NC group. Error bars represent ± SD. Comparisons between groups analyzed by Student's *t*‐test. *p < 0.05, **p < 0.01, ***p < 0.001. DEGs, differentially expressed genes; NC, negative control; SAT, subcutaneous adipose tissue; si‐Usp34, small interfering RNA targeting Usp34; SI‐Exos, small intestinal epithelial exosomes; SVF, stromal vascular fraction.

We identified 10 miRNAs consistently upregulated in both SI‐Exos and serum exosomes from young (3‐month‐old) compared to aged (18‐month‐old) mice (GEO: GSE283891, Figure 4A) (He et al. 2025). To identify the miRNA critical for PDGFRα^+^ progenitor cells, we measured the expression levels of these 10 miRNAs in SI‐Exos, SAT, and SVF from young (3‐month‐old) and aged (20‐month‐old) mice. Compared to aged mice, miR‐379‐5p expression was significantly higher in SI‐Exos, SAT, and SVF of young mice (Figure 4B). Neither SAT nor SVF expressed pre‐miR‐379. These results indicated that miR‐379‐5p in young exosomes might enhance lipid storage in PDGFRα^+^ progenitor cells.

Next, we examined the effect of miR‐379‐5p inhibition on lipid droplet accumulation in PDGFRα^+^ progenitor cells from SAT. Using a miR‐379‐5p inhibitor (52% knockdown efficiency, Figure 4C), genes involved in adipogenesis, fatty acid and triglyceride synthesis, lipid droplet formation/fusion, and lipolysis were downregulated (Figure S3C). Following adipogenic induction, miR‐379‐5p knockdown significantly reduced lipid droplet accumulation (Figure 4D). Conversely, overexpression of miR‐379‐5p enhanced adipocyte differentiation, lipid metabolism, and lipid droplet formation (Figure 4E,F, Figure S3D). These findings suggested that miR‐379‐5p in young exosomes augmented lipid droplet accumulation in PDGFRα^+^ progenitor cells.

To determine whether miR‐379‐5p in SI‐Exos directly promotes lipid storage, we transfected SI‐Exos from aged mice (20m‐SI‐Exos) with miR‐379‐5p mimics or negative control (NC) and co‐cultured them with PDGFRα^+^ progenitor cells from SAT. Overexpression of miR‐379‐5p in 20m‐SI‐Exos promoted adipocyte differentiation, lipid metabolism, and lipid droplet formation, significantly increasing lipid droplet accumulation (Figure 4G,H, Figure S3E).

To identify miR‐379‐5p target genes, RNA‐seq was performed on PDGFRα^+^ progenitor cells transfected with miR‐379‐5p inhibitor or NC. Analysis revealed 379 upregulated and 250 downregulated DEGs (Figure 4I). The top 100 upregulated and 100 downregulated DEGs were visualized in volcano plots (Figure 4J). By intersecting database predictions (miRWalk, TargetScan, and TarBase) with the top DEGs, six candidate target genes related to lipid metabolism, Usp34, Atm, Mtf1, Stab1, Klf12, and Fbn2, were identified (Figure 4J). RT‐qPCR validation confirmed significant downregulation of Usp34, Atm, and Mtf1 after transfection with miR‐379‐5p mimics (Figure 4K). To validate their functional relevance, siRNA‐mediated knockdown of Usp34, Atm, and Mtf1 was performed in PDGFRα^+^ progenitor cells. Only Usp34 silencing significantly upregulated lipogenic markers (PPARγ, ACLY, and CIDEC) (Figure 4L,M), while Atm knockdown showed no effect, and Mtf1 depletion had opposite effects (Figure S5A–D). GO analysis revealed that Usp34 and related genes (including Dvl3, Apc, Ncoa3, Macf1) were involved in the Wnt signaling pathway (Figure 4N). These results indicated that miR‐379‐5p promotes lipid droplet formation in PDGFRα^+^ progenitor cells via Usp34.

### 2.5 Exosomal miR‐379‐5p Directly Targeted Usp34 via Wnt/β‐Catenin to Regulate Lipid Droplet Formation in PDGFRα ^+^ Progenitor Cells

**FIGURE 5:**
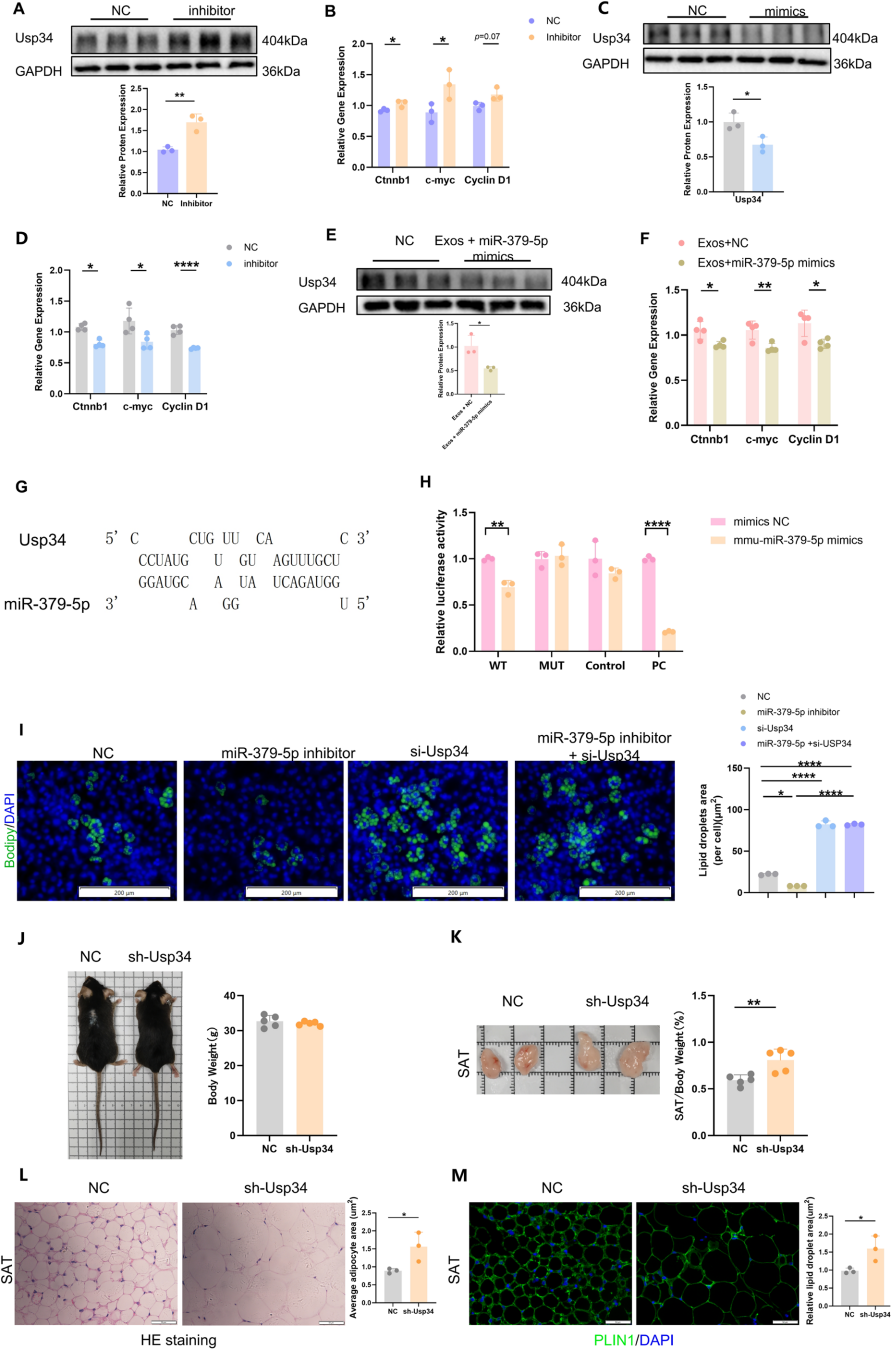
Exosomal miR‐379‐5p directly targeted Usp34 via Wnt/β‐catenin. (A, B) PDGFRα^+^ progenitor cells from SAT transfected with miR‐379‐5p inhibitor or negative control (NC). (A) Usp34 protein levels at 72 h (*n* = 3). (B) Relative mRNA levels of Ctnnb1, c‐myc, and Cyclin D1 at 48 h (*n* = 3). (C‐D) PDGFRα^+^ progenitor cells transfected with miR‐379‐5p mimics or NC. (C) Usp34 protein levels at 72 h (*n* = 3). (D) Relative mRNA levels of Ctnnb1, c‐myc, and Cyclin D1 at 48 h (*n* = 4). (E‐F) SI‐Exos from aged (20‐month‐old) mice transfected with miR‐379‐5p mimics or NC and co‐cultured with PDGFRα^+^ progenitor cells. (E) Usp34 protein levels at 72 h (*n* = 3). (F) Relative mRNA levels of Ctnnb1, c‐myc, and Cyclin D1 at 48 h (*n* = 4). (G) Sequence alignment showing miR‐379‐5p binding site in Usp34 3′‐UTR. (H) Dual‐luciferase assay confirming miR‐379‐5p directly targets Usp34 in HEK293T cells at 48 h (*n* = 3). Relative activity normalized to Renilla/Firefly (RLUC/FLUC). (I) PDGFRα^+^ progenitor cells transfected with NC, miR‐379‐5p inhibitor, si‐Usp34, or miR‐379‐5p inhibitor plus si‐Usp34, followed by 6‐day adipogenic induction. Lipid droplets visualized by BODIPY (green) and DAPI (blue). Scale bar, 200 μm (*n* = 3). (J‐M) Usp34 knockdown in 20‐month‐old mice via local AAV injection into inguinal SAT. Mice received AAV‐sh‐Usp34 or AAV‐NC and were analyzed after 2 weeks (*n* = 5). (J) Representative mouse images and body weight. (K) SAT images and weight (*n* = 5). (L) H&E staining of adipocytes in SAT (*n* = 3). Scale bar, 50 μm. (M) PLIN1 (green, lipid droplet marker) and DAPI (blue, nuclei) immunofluorescence in SAT (*n* = 3). Scale bar, 50 μm. Error bars represent ± SD. Comparisons analyzed by Student's t‐test. *p < 0.05, **p < 0.01, ***p < 0.001, ****p < 0.0001. SAT, subcutaneous adipose tissue; SI‐Exos, small intestinal epithelial exosomes; NC, negative control; sh‐Usp34, short hairpin RNA targeting Usp34; si‐Usp34, small interfering RNA targeting Usp34.

To test this hypothesis, we evaluated the expression of Usp34 and key components of the Wnt/β‐catenin pathway (Ctnnb1, c‐myc, and Cyclin D1) in PDGFRα^+^ progenitor cells isolated from mouse SAT following miR‐379‐5p overexpression or inhibition. Inhibition of miR‐379‐5p increased Usp34 protein expression (Figure 5A) and elevated mRNA levels of Ctnnb1, c‐myc, and Cyclin D1 (Figure 5B). Conversely, miR‐379‐5p overexpression reduced expression of Usp34, Ctnnb1, c‐myc, and Cyclin D1 (Figure 5C,D). Similar results were observed when PDGFRα^+^ progenitor cells were treated with 20m‐SI‐Exos overexpressing miR‐379‐5p (Figure 5E,F). A luciferase reporter assay confirmed that miR‐379‐5p directly targeted the 3′‐UTR of Usp34 (Figure 5G,H). These findings suggested that exosomal miR‐379‐5p suppressed Usp34 expression in PDGFRα^+^ progenitor cells, subsequently downregulating Wnt/β‐catenin signaling to promote adipocyte differentiation, lipid droplet formation, and lipid metabolism in mouse SAT.

To further confirm that miR‐379‐5p promoted lipid droplet accumulation via Usp34, a rescue experiment was performed. PDGFRα^+^ progenitor cells were co‐transfected with miR‐379‐5p inhibitor and si‐Usp34, and lipid droplet formation was assessed. Usp34 knockdown efficiency ranged from 1.77‐ to 1.79‐fold (Figure S4E,F). Inhibition of miR‐379‐5p reduced expression of ACLY, DGAT1, and CIDEC and decreased total lipid droplet area per nucleus (Figure 5I, Figure S5G). However, simultaneous Usp34 knockdown reversed these effects, increasing expression of ACLY, DGAT1, and FIT2 and restoring lipid droplet accumulation (Figure 5I, Figure S5G). These results indicated that the suppression of adipogenesis caused by miR‐379‐5p inhibition could be rescued by Usp34 knockdown.

In PDGFRα^+^ progenitor cells, Usp34 knockdown reduced both total and active (non‐phosphorylated) β‐catenin protein expression (Figure S6A). Dual‐luciferase reporter assays showed significantly decreased β‐catenin/TCF transcriptional activity (Figure S6B). Following adipogenic induction, β‐catenin was observed in both cytoplasm and nucleus, but its abundance in both compartments was markedly reduced by Usp34 silencing (Figure S6C).

To validate Usp34's role in vivo, a short hairpin RNA adeno‐associated virus targeting Usp34 (AAV‐sh‐Usp34) was injected into the inguinal SAT of 20‐month‐old mice. Body weight remained unchanged, while SAT weight significantly increased (Figure 5J,K). HE staining and PLIN1 immunofluorescence demonstrated increased adipocyte size in SAT (Figure 5L,M). Thus, miR‐379‐5p regulates adipogenesis in PDGFRα^+^ progenitor cells of SAT by targeting Usp34 and suppressing Wnt/β‐catenin signaling.

### 2.6 Single‐Cell Analysis Revealed That Young SI‐Exos Remodel Adipose Tissue by Regulating Progenitor Cell States

**FIGURE 6:**
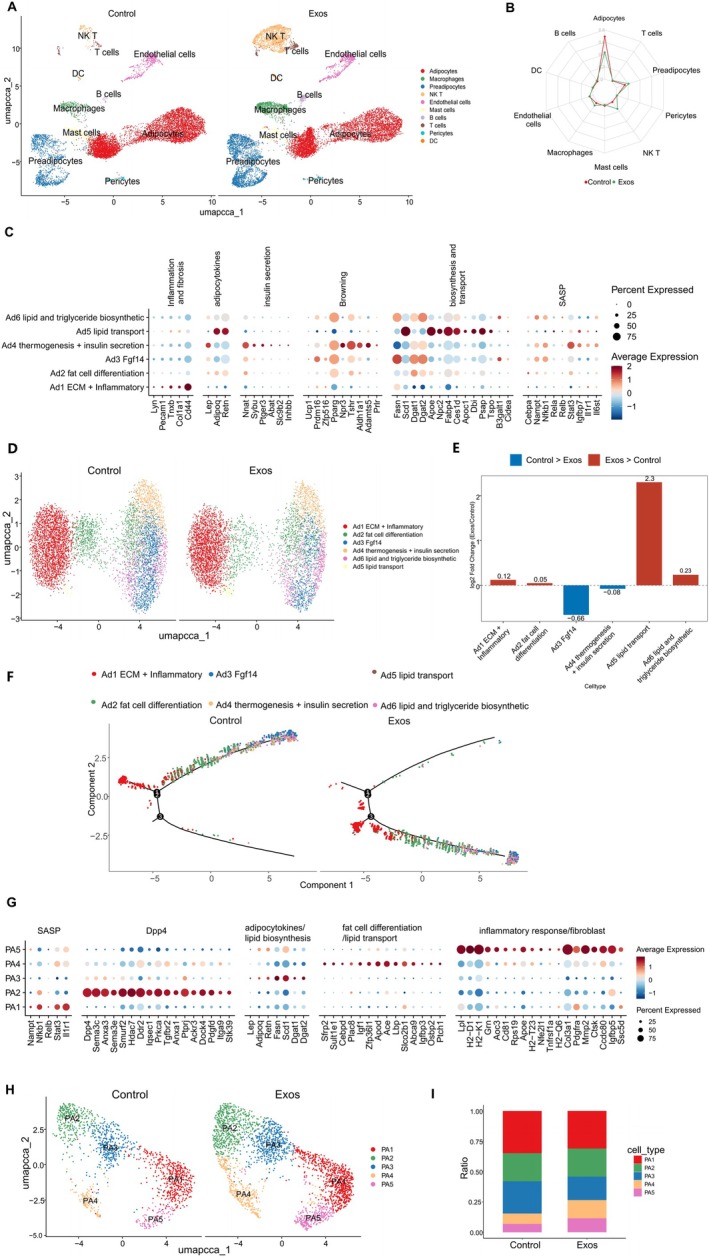
scRNA‐seq analysis of SAT. scRNA‐seq of SAT from aged mice treated via tail vein injection with PBS or 3m‐SI‐Exos (20 injections, every 3 days for 2 months). (A) UMAP visualization of all SAT cells colored by treatment group (3m‐SI‐Exos vs. PBS). (B) Radar chart comparing cell‐type composition between PBS and 3m‐SI‐Exos‐treated SAT. (C) Dot plot illustrating functional states of adipocyte subclusters. (D) UMAP visualization of adipocytes. (E) Proportions of adipocyte subpopulations (AD1‐AD6) between groups. (F) Pseudotime trajectory analysis of adipocytes. (G) Dot plot illustrating functional states of preadipocyte subclusters. (H) UMAP visualization of preadipocytes. (I) Proportions of preadipocyte subpopulations (PA1‐PA5) between groups. SAT, subcutaneous adipose tissue; 3m‐SI‐Exos, small intestinal epithelial exosomes from 3‐month‐old mice.

Adipose tissue is a highly plastic organ composed of various cell types, and its aging involves inflammation and structural remodeling (Zhao et al. 2024). To comprehensively characterize changes in adipocyte subpopulations, scRNA‐seq was performed on SAT from aged mice intravenously injected with 3m‐SI‐Exos. Ten major cell types were identified and annotated using known markers (Figure 6A). Comparative analysis revealed that exosome treatment reduced the proportion of mature adipocytes but increased preadipocyte populations (Figure 6B). Given that adipocytes represented the most abundant and dynamically changing cells, they were further divided into six subclusters based on highly expressed genes: AD1 (extracellular matrix and inflammation), AD2 (adipocyte differentiation), AD3 (Fgf14‐associated), AD4 (thermogenesis and insulin secretion), AD5 (lipid transport), and AD6 (lipid and triglyceride synthesis) (Figure 6C). Notably, the proportions of AD5 and AD6 subpopulations significantly increased in the 3m‐SI‐Exos group, suggesting enhanced lipid transport and synthesis (Figure 6D,E). Preadipocytes were classified into five subsets: PA1 (SASP‐related), PA2 (dipeptidyl peptidase‐4 (DPP4)‐high), PA3 (adipokines and lipid synthesis), PA4 (adipocyte differentiation and lipid transport), and PA5 (inflammation and fibroblast‐like) (Figure 6G). The PA4 subset markedly increased after exosome treatment (Figure 6H,I).

Consistent with these findings, exosome‐treated adipocytes primarily localized to the lower branch of the pseudotime trajectory, while control cells clustered in the upper branch. This suggested promotion of AD5 and AD6 subsets by SI‐Exos (Figure 6F). Further developmental pseudotime analysis combining preadipocytes and adipocytes indicated no significant differences after exosome treatment, implying normal preadipocyte differentiation (Figure S7A). Collectively, these data suggested that SI‐Exos influenced lipid droplet transport and synthesis without affecting overall differentiation processes.

Notably, immune cells changed significantly, particularly NK cells (Figure 6A,B). This suggested an impact of intestinal exosomes on the immune microenvironment. RT‐qPCR revealed reduced expression of inflammatory markers in the 3m‐SI‐Exos group (Figure S8A). Single‐cell analysis of SAT macrophages identified four subpopulations: anti‐inflammatory macrophages (Anti‐M), pro‐inflammatory macrophages (Pro‐M), resident tissue macrophages (RTM), and lipid‐associated macrophages (LAM) (Figure S8B,C).

Beyond morphological changes, SI‐Exos induced distinct functional reprogramming: SAT adopted an anti‐inflammatory, energy‐storage phenotype, whereas VAT transitioned toward lipid mobilization. These divergent outcomes were supported by GO enrichment analyses of scRNA‐seq data (Figure S9). Additionally, scRNA‐seq analysis revealed differential expression enrichment in muscle‐related pathways, including myofiber differentiation, myotube fusion, and neurovascular regulation (Figure S7B).

## 3 Discussion

With the rapid aging of the global population, there is an increasing need to clarify how SAT atrophy influences metabolic dysfunction and internal organ disorders. Understanding adipose tissue aging could offer novel strategies to delay systemic aging. Here, we reported miRNA high‐throughput sequencing to characterize age‐related changes in SI‐Exos. Through combined animal and in vitro experiments, we demonstrated how young SI‐Exos reversed SAT atrophy in aged mice and identified molecular pathways driving lipid droplet formation in PDGFRα^+^ progenitor cells of SAT.

Firstly, we showed that SI‐Exos and their miRNA cargo underwent significant age‐dependent alterations, consistent with previous studies (He et al. 2025). High‐throughput sequencing revealed that aging increased 30 miRNAs and decreased 102 miRNAs. These miRNAs primarily participated in biological processes such as adipocyte differentiation, lipid synthesis and storage, and inflammation. Exosomes secreted by the small intestine can enter systemic circulation and mediate remote regulatory effects (Feng et al. 2023). Thus, considering the miRNAs consistently regulated in intestinal and serum exosomes, we proposed an important role for SI‐Exos in regulating adipose metabolism during aging.

Adipose tissue, crucially involved in metabolic dysfunction‐related diseases, undergoes remodeling during aging. Aging redistributes adipose tissue, characterized by decreased SAT and increased VAT (Palmer and Kirkland 2016). In our study, aged mice injected with young SI‐Exos showed enhanced grip strength, improved endurance, reduced alopecia, reversal of SAT atrophy, promotion of browning in SAT and VAT, and decreased inflammation in adipose tissues. Young exosomes promoted lipid synthesis and accelerated lipid utilization (lipolysis). Previous studies indicated that adipocyte size and lipid turnover significantly affect systemic insulin sensitivity (Arner et al. 2019; Spalding et al. 2017). Thus, increased lipid turnover might prevent metabolic stagnation common in aged SAT and reflect improved tissue vitality (Arner et al. 2011). Consistent with these phenotypes, RNA‐seq identified 83 upregulated and 1184 downregulated DEGs in the 3m‐SI‐Exos group. These genes were mainly enriched in pathways related to lipid synthesis, lipid transport, inflammation, and apoptosis. For the first time, we highlighted that young SI‐Exos reversed SAT atrophy and reduced inflammation in VAT.

VAT promotes inflammation, whereas SAT has a more complex function. Thus, profiling and distinguishing adipose subtypes are critical for understanding aging‐related SAT changes. Hagberg and Spalding (2024) and Maniyadath et al. (2023) showed that PDGFRα^+^ progenitor cells are major sources of new adipocytes in WAT, with potential for both beige and white adipocyte differentiation (Hagberg and Spalding 2024; Maniyadath et al. 2023). Specifically, PDGFRα expression predominantly localized to preadipocytes, with a marked increase in PDGFRα^+^ progenitor cells observed in the 3m‐SI‐Exos group. Through in vitro experiments, we confirmed that young SI‐Exos enhanced adipocyte differentiation, lipid droplet formation, and lipid metabolism in PDGFRα^+^ progenitor cells, significantly increasing lipid accumulation. These findings suggest that young SI‐Exos reverse age‐related SAT atrophy by promoting lipid synthesis in PDGFRα^+^ progenitor cells.

To elucidate the underlying mechanisms, we performed high‐throughput sequencing of exosomal miRNAs and identified miR‐379‐5p as a key regulator of lipid synthesis and lipid droplet dynamics. Previous research indicated that miR‐379‐5p modulates cholesterol metabolism via the STAT1/HMGCS axis (Dong et al. 2022), reduces lipotoxicity, and ameliorates hypertriglyceridemia (de Guia et al. 2015), but its role in SAT remained unknown. Our knockdown and overexpression experiments confirmed that miR‐379‐5p promoted lipid droplet synthesis. Bioinformatics analysis identified six potential target genes, including Usp34, a deubiquitinating enzyme that negatively regulates Wnt/β‐catenin signaling by stabilizing Axin (Deng et al. 2020; Lui et al. 2011; Park et al. 2020; Ross et al. 2000). Consistent with this, our RNA‐seq GO analysis implicated Usp34 in the Wnt pathway, making it a likely mediator of miR‐379‐5p's regulation of lipid droplet formation. Dual‐luciferase assays confirmed miR‐379‐5p directly targeted Usp34's 3′‐UTR, inhibiting its expression. Functional experiments demonstrated that Usp34 inhibition significantly promoted lipid droplet formation and adipose expansion both in vitro and in vivo. Collectively, these results indicate that miR‐379‐5p from young SI‐Exos enhances lipid droplet synthesis in PDGFRα^+^ progenitor cells of aged SAT by inhibiting Usp34 and downregulating Wnt/β‐catenin signaling.

Adipose tissue is the first organ to exhibit age‐related transcriptomic changes beginning from middle age. Recent scRNA‐seq studies identified over 60 distinct cellular subpopulations within adipose tissue, including adipocytes, adipose progenitor cells, fibroblasts, vascular cells, and immune cells (Massier et al. 2023; Yang et al. 2024). Our scRNA‐seq analysis of SAT revealed six adipocyte subpopulations, among which the AD5 and AD6 subsets showed pronounced lipid droplet formation. Besides promoting a lipogenic phenotype in SAT, SI‐Exos administration significantly increased SAT progenitor cell numbers. Although the differentiation hierarchy and regulatory mechanisms of adipocyte progenitors remain unclear, we further classified preadipocytes into five subsets. Notably, the PA2 subpopulation specifically expressed dipeptidyl peptidase‐4 (DPP4). Merrick et al. (2019) reported that cells expressing DPP4/CD26 are highly proliferative and multipotent progenitors (Merrick et al. 2019). Regarding the observed differences in preadipocyte and adipocyte numbers, pseudotime analysis showed no significant differences in preadipocyte‐to‐adipocyte differentiation after exosome treatment. Furthermore, in the exosome‐treated group, subcutaneous adipocyte size and total SAT volume significantly increased. Thus, we propose that increased adipocyte size may have reduced the efficiency of cell capture during single‐cell library preparation, resulting in fewer cells detected per unit volume.

The adipose tissue microenvironment also significantly influences adipocyte fate. For example, Xiaotong Yu et al. highlighted that macrophages expressing LYVE1 regulate adipose stem cell differentiation and tissue expansion (Yu et al. 2025). We observed a significant increase in immune cells, particularly NK cells, in SAT following intestinal exosome treatment. Interestingly, recent studies in centenarians suggested a youthful NK cell profile might be central to healthy aging and longevity (Wang, Zhang, et al. 2025). Collectively, these findings imply that intestinal exosomes enhance preadipocyte differentiation and modulate the immune microenvironment, promoting SAT maintenance during aging.

Notably, young SI‐Exos reversed SAT atrophy and significantly reduced VAT. Accumulating evidence suggests SAT exerts beneficial metabolic effects. Thus, increased SAT volume likely contributed to reduced VAT. Additionally, aged mice treated with young SI‐Exos showed improved muscle strength. We propose several possible explanations. First, expanded SAT may reduce intramuscular fat deposition, enhancing muscle quality. Second, FAPs located in SAT can migrate into skeletal muscle, supporting muscle repair (Sastourné‐Arrey et al. 2023). Third, scRNA‐seq analysis revealed differentially expressed genes enriched in muscle‐related pathways, such as myofiber differentiation, myotube fusion, and neurovascular regulation. Furthermore, FAPs in skeletal muscle and adipose tissue are both PDGFRα^+^ cells (Dani and Pfeifer 2017; Giuliani et al. 2022). Therefore, direct uptake of young SI‐Exos by skeletal muscle and VAT cannot be excluded. While our study primarily focused on SAT PDGFRα^+^ progenitors, further investigations are necessary to clarify the broader physiological impacts of these exosomes.

A bidirectional regulatory relationship between gut‐derived exosomes and microbiota has recently attracted significant attention (Shen et al. 2022; Yan et al. 2023). For instance, exosomal miR‐30a‐5p expression inhibits Lactobacillus growth (Li et al. 2025), while *Fusobacterium nucleatum* significantly elevates miR‐129‐2‐3p levels in intestinal epithelial exosomes (Wei et al. 2023). To assess whether the gut microbiota regulates age‐associated SI‐Exos changes, we established a fecal microbiota transplantation model. However, changes in exosomal profiles after fecal transplantation from 3‐month‐old and 20‐month‐old mice into germ‐free mice did not match age‐related alterations. For the first time, we highlight that, although intestinal microbiota influences SI‐Exos regulation, age‐related changes in exosome profiles are not directly microbiota dependent.

While our findings emphasize a critical role of SI‐Exos in mitigating age‐related SAT atrophy, several limitations remain. First, the human relevance of these findings is unclear, as all experiments were conducted in mice. Future studies should investigate the miR‐379‐5p/Usp34 pathway in human adipose tissue. Second, the precise mechanisms controlling miR‐379‐5p packaging into intestinal exosomes and its selective secretion into circulation remain unknown. Finally, the long‐term safety and efficacy of exosome‐based therapies must be thoroughly evaluated prior to clinical translation.

In conclusion, our study systematically elucidates the mechanism by which young SI‐Exos promote lipid droplet formation in PDGFRα^+^ progenitor cells via the miR‐379‐5p/Usp34/Wnt axis, reversing age‐related SAT atrophy and reducing visceral obesity. These findings not only deepen understanding of the gut–adipose axis in aging but also provide novel insights and potential therapeutic targets for age‐related metabolic diseases.

## 4 Methods

### 4.1 Mice

Male C57BL/6J (B6) mice were purchased from Nanjing Medical University. The mice were housed in a controlled environment at 22°C ± 4°C, 60% relative humidity, and a 12‐h light/dark cycle (light: 08:00–20:00; dark: 20:00–08:00). All animal protocols were approved by the Animal Ethical and Welfare Committee of Nanjing Medical University (IACUC‐240723, IACUC‐240723‐1). Animals were provided appropriate care, and experiments followed institutional guidelines.

### 4.2 Isolation of Exosomes From SI

Isolated small intestines were cut into 4–5 cm segments, everted, and washed with 10 mmol/L DTT in ice‐cold PBS. After rinsing with PBS, segments were incubated in ice‐cold chelation buffer (8 mM EDTA‐Na_2_) for 30 min. Tissues were then vibrated in PBS, and the resulting suspension was centrifuged at 3000 rpm for 5 min at 4°C. Dead cells and debris were removed by subsequent centrifugation at 300 × g for 10 min. The supernatant was filtered (0.22 μm), transferred to polycarbonate tubes, and ultracentrifuged at 100,000 × *g* for 2.5 h at 4°C. Exosome pellets were washed, resuspended in ice‐cold PBS according to pellet size, stored at −80°C, and resuspended before use.

### 4.3 PKH26 Labeling and Exosome Treatment In Vivo/In Vitro

Exosomes were labeled using the PKH26 Red Fluorescent Cell Linker Kit (Sigma‐Aldrich, Cat#MIDI26). Briefly, 100 μg exosomes were diluted in 1 mL Diluent C, mixed with 4 μL PKH26, and incubated for 5 min. The labeling reaction was stopped by adding 2 mL 1% exosome‐depleted FBS, followed by ultracentrifugation (100,000 × *g*, 20 min, 4°C). The labeled exosome pellet was resuspended in 100 μL PBS. For in vivo tracking, labeled exosomes from intestinal epithelium were injected into C57BL/6J mice. Tissues were harvested after 24 h and imaged using an IVIS Spectrum system. For in vitro assays, exosomes were incubated with 10^5^ cells for 24 h, and fluorescence was visualized under an Olympus microscope.

### 4.4 MiRNA High‐Throughput Sequencing

Small intestinal exosomal RNA was extracted from male mice at 3, 8, 15, and 18 months of age for miRNA sequencing. After quality control, 100 ng total RNA was used for library preparation with the VAHTS Small RNA Library Prep Kit, utilizing the unique 3′‐uridine and 5′‐phosphate structures of small RNAs. Adaptors were ligated, followed by cDNA synthesis, PCR amplification, and purification. Libraries with inserts of 140–160 bp were selected using PAGE or magnetic beads. Library quality was assessed using Qubit 3.0 and Agilent 2100 Bioanalyzer, and concentration was quantified by qPCR (> 2 nM). Qualified libraries were pooled and sequenced on a HiSeq platform in SE50 mode.

### 4.5 Single‐Cell Data Processing

Single‐cell RNA‐seq raw reads were aligned and mapped to the mm10 genome using the CellRanger (v7.2.0) pipeline with default parameters. Processed data were imported into the R package Seurat (v5.1.0) using parameters (min.cells = 3, min.features = 200). Cells expressing < 500 or > 6000 genes or > 10% mitochondrial genes were filtered out. After filtering, 21,844 cells remained. Gene expression data were normalized using default Seurat parameters. The top 2000 highly variable genes (HVGs) were identified with the FindVariableFeatures function, followed by scaling of the expression matrix. Principal component analysis (PCA) was performed for dimensionality reduction. To correct for batch effects between samples, data integration was conducted using the IntegrateLayers function with the CCAIntegration algorithm based on the top 30 PCs.

### 4.6 Clustering and Cell Type Identification by scRNA‐Seq

Cell clusters were identified using FindClusters (resolution = 1.2) in Seurat, and visualized by Uniform Manifold Approximation and Projection (UMAP). Unsupervised clustering produced 25 cell clusters. Cell markers were identified from existing literature and the CellMarker database (https://xteam.xbio.top/CellMarker/). Cell clusters were annotated according to identified markers, and the distribution and proportion of each cell type were evaluated. To explore the heterogeneity of adipocytes and preadipocytes, these cells were reclustered separately, and each subpopulation was annotated based on distinct gene expression signatures.

### 4.7 Analysis of Adipocyte and Preadipocyte Subpopulations

Pseudotime trajectories for adipocyte and preadipocyte subpopulations were reconstructed using Monocle2 (v2.22.0) with the DDRTree algorithm. Cells were ordered along pseudotime to track developmental transitions. Cell–cell communication was analyzed using the R package CellChat (v2.12) with default parameters, employing the CellChatDB mouse database. Each sample was analyzed separately, followed by comparative analysis.

### 4.8 Enrichment Analysis of DEGs

DEGs in adipocyte and preadipocyte subpopulations were identified using the FindAllMarkers function (Wilcoxon rank‐sum test, default parameters). The FindMarkers function was used to compare cell subpopulations between control and exosome‐treated samples. Genes with log2FoldChange > 1 and adjusted *p*‐value (*p*adj) < 0.05 were defined as DEGs. GO and KEGG enrichment analyses of DEGs were performed using the clusterProfiler package. Results were visualized using ggplot2 functions.

### 4.9 Fecal Microbiota Transplantation

Eight‐week‐old germ‐free C57BL/6J mice were intragastrically administered fecal microbiota from 3‐month‐old and 20‐month‐old donor mice or PBS control. The treatment was performed twice (200 mg per dose) within 1 week. After successful colonization, mice were maintained for one additional week, anesthetized, and euthanized by cervical dislocation.

### 4.10 Fluorescence‐Activated Cell Sorting

Cells (~3 × 10^7^ cells/mL) were incubated with primary antibodies for 30 min at 4°C in PBS containing 2 mM EDTA and 2% FBS. Primary antibodies included anti‐Sca‐1 (1:100–400) and anti‐PDGFRα (1:50–200). Fluorescence minus one (FMO) controls with appropriate isotype antibodies were used. Cells were resuspended at approximately 1 × 10^7^ cells/mL and analyzed using an LSRII instrument (FACSDiva software). Sorting gates were defined based on isotype controls, and data analysis utilized biexponential transformation (FlowJo v10.8.1).

### 4.11 Isolation of SVF, PDGFRα ^+^ Progenitor Cells, and Adipogenic Differentiation

Adipose progenitor cells were isolated from SAT. Tissues were digested with 10 mg/mL collagenase II (Sigma‐Aldrich, Cat#C6885‐5G), filtered, and the SVF was collected by centrifugation. After red blood cell lysis, PDGFRα^+^ progenitors were isolated using PE‐conjugated antibodies and magnetic bead selection (Invitrogen, Cat#12‐1401‐81). Cells positive or negative for PDGFRα were separated using coated magnetic beads (PE Selection Kit, Stem Cell, Cat#17666), followed by positive selection with an APC Selection Kit (Stem Cell, Cat#17667). Purified PDGFRα^+^ progenitors were cultured in DMEM with 15% exosome‐depleted fetal bovine serum (SBI System Biosciences, Cat#Exo‐FBS‐50A‐1). Cells were induced to differentiate with DMEM containing 15% exosome‐depleted FBS, 0.5 mM IBMX (Sigma‐Aldrich, Cat#I5879‐1G), 20 nM insulin, and 0.25 μM dexamethasone for 48 h, followed by 20 nM insulin for an additional 48 h, and then maintained in DMEM with 15% exosome‐depleted FBS.

### 4.12 Hematoxylin‐Eosin Staining

Tissue sections were dewaxed, hydrated, stained with hematoxylin (3 min), rinsed with water, differentiated with acid ethanol (5 s), rinsed again (5 min), counterstained with eosin (2 min), dehydrated, cleared with xylene, and mounted. Imaging was performed with an Olympus DP72 microscope (Olympus Corporation, Japan).

### 4.13 Immunofluorescence Staining

After dewaxing, hydration, and antigen retrieval in citric acid buffer (95°C–99°C, 20 min), sections were permeabilized and blocked, then incubated overnight at 4°C with PLIN1 (Proteintech, Cat#27716‐1‐AP) and UCP1 (Proteintech, Cat#23673‐1‐AP). After PBS washes, sections were incubated with secondary antibodies, washed again, counterstained with DAPI (Cell Signaling Technology, Cat#4083S), and mounted.

### 4.14 Bodipy Staining

Differentiated FAPs were washed with PBS, fixed in 4% paraformaldehyde for 30 min, washed again, and stained with Bodipy (HY‐W090090, USA) for 30 min. Cells were washed, counterstained with DAPI (Cell Signaling Technology, Cat#4083S) for 5 min, washed again, and mounted.

### 4.15 miRNA Mimics/Inhibitor Transfection and Exosome Transfections

When FAPs reached 70%–80% confluence, miR‐379‐5p mimics, miR‐379‐5p inhibitors, or Usp34 siRNA were transfected using Lipofectamine 3000 (eBioscience, Cat#100022052) according to the manufacturer's instructions. MiRNA mimics, inhibitors, siRNA for ctnnb1, and corresponding negative controls were purchased from GenePharma (Shanghai, China). The medium was replaced with DMEM growth medium 24 h after transfection.

Exosome transfection was performed using Exo‐Fect Exosomes Transfection Reagent (SBI System Biosciences, Cat#EXFT20A‐1) following the manufacturer's guidelines. For fluorescent siRNA transfection, 50 μg aged exosomes were transfected with 370 pmol fluorescent siRNA.

### 4.16 Luciferase Reporter Assay

Luciferase reporter plasmids (GP‐miRGLO) containing wild‐type (WT‐Usp34) or mutant (Mut‐Usp34) 3′UTR of Usp34 were constructed by GenePharma (Shanghai, China). HEK293T cells seeded in 48‐well plates were co‐transfected with luciferase reporter plasmids (WT or Mut), miR‐379‐5p mimics, or negative control using X‐tremeGENE HP DNA Transfection Reagent. Cells were harvested 24 h post‐transfection, and luciferase activity was measured with the Dual‐Glo Luciferase Assay System (GenePharma) following the manufacturer's instructions. Firefly luciferase served as a normalization control.

### 4.17 RNA Extraction and Quantitative Real‐Time PCR

Total RNA was extracted using TRIzol reagent (Invitrogen, Cat#15596026CN) according to the manufacturer's instructions. cDNA synthesis used a reverse transcription kit (TaKaRa, Japan). Quantitative PCR utilized SYBR Green master mix (Vazyme, Nanjing, China) on a StepOne Real‐Time PCR System (Applied Biosystems, USA). Primer sequences are listed in Table S1. The 2^−ΔΔCT^ method was used for data analysis.

### 4.18 Protein Extraction and Western Blot Analysis

Cells were lysed on ice for 30 min in RIPA buffer (KeyGEN, Nanjing, China, Cat#KGB5203‐100) with protease inhibitors (Cat#KGB5101‐100). Lysates were centrifuged (12,000 rpm, 10 min, 4°C) and supernatants collected. Protein samples were separated by 10% SDS‐PAGE, transferred to PVDF membranes (Millipore, USA), blocked (5% skim milk, 2 h), incubated with primary antibodies overnight at 4°C, followed by secondary antibodies (2 h, room temperature). Bands were visualized using chemiluminescence and quantified using Image Lab software. Primary antibodies included GAPDH (1:5000, Bioworld, Cat#AP0063), FASN (1:5000, Proteintech, Cat#66591‐1‐Ig), ACLY (1:1000, Proteintech, Cat# 67166591‐1‐Ig), PPARγ (1:5000, Proteintech, Cat#66936–1‐Ig), PLIN1 (1:1000, Affinity, Cat#DF7602), and Usp34 (1:1000, Affinity, Cat# DF9990).

### 4.19 Statistical Analysis

Data are presented as mean ± SD. Two‐tailed Student's *t*‐tests were used for pairwise comparisons, and one‐way ANOVA was used for multiple group comparisons. Analyses were performed using GraphPad Prism 10.0. Statistical significance levels were set at: *p < 0.05, **p < 0.01, ***p < 0.001.

## Author Contributions

S.L., Y.F.L. and G.X.D. designed the study, analyzed and interpreted the data, and prepared the paper. T.T.H. performed the experiments, interpreted the data and wrote the manuscript. Y.H. performed biostatistical analysis of the single‐cell RNA‐seq datasets. Y.Z. analyzed RNA‐seq; Y.L.S. was responsible for miRNA high‐throughput sequencing. L.J.Y. provided histological analysis. X.B.L. performed the primary pre‐adipocyte progenitor isolation and adipocyte differentiation. J.Y. and F.X. provided technical help. All authors have read and approved the manuscript.

## Funding

This study was funded by the National Key R&D Program of China (2022YFA0806103 to G.X.D), National Natural Science Foundation of China (82471588 to G.X.D., 82201740 to Y.F.L.), Natural Science Foundation of Jiangsu Province (BK20241787 to S.L.), Development Fund of the Affiliated Hospital of Xuzhou Medical University(ZX202402 to S.L., ZX202407 to Y.H.).

## Conflicts of Interest

The authors declare no conflicts of interest.

## Supporting information


**Figure S1:** Comprehensive characterization of exosome preparations. (A) Transmission electron microscopy showing typical cup‐shaped morphology with a diameter of approximately 100 nm, consistent with exosome ultrastructure. (B) Nanoparticle tracking analysis revealing a size distribution peak between 100 and 120 nm. (C) Western blot analysis demonstrating enrichment of exosome markers (GPA33, TSG101, and CD63) and the absence of cellular contamination marker (Calnexin). Scale bar, 100 nm. Exos, exosomes.
**Figure S2:** Effects of fecal microbiota transplantation on adipose tissue. Eight‐week‐old germ‐free C57BL/6J mice received FMT from young (3‐month‐old) or aged (20‐month‐old) donors mice, or PBS, twice (200 mg/dose) in 1 week. Subcutaneous adipose tissue was analyzed after one additional week. (A) Representative images and body weights of mice after transplantation. (B) Adipose tissue morphology and weights of SAT and VAT (*n* = 3). (C) H&E staining of adipocytes from SAT and VAT (*n* = 3). Scale bar, 50 μm. Error bars represent ± SD. Comparison between two groups was performed by Student's t‐test. *p < 0.05, **p < 0.01, ***p < 0.001. SI‐Exos, small intestinal epithelial exosomes; SAT, subcutaneous adipose tissue; VAT, visceral adipose tissue.
**Figure S3:** Relative gene expression of lipogenesis‐related genes. (A) 20‐month‐old mice received tail‐vein injections of SI‐Exos derived from 3‐month‐old mice (100 μg per injection, every 3 days for 2 months, total 20 injections). Control mice received an equal volume of PBS. Gene expression related to adipocyte differentiation, triglyceride and fatty acid synthesis, lipid droplet formation, and lipolysis (*n* = 3). (B) PDGFRα+ and PDGFRα− progenitor cells were isolated using magnetic beads and induced to undergo adipogenesis for 6 days. Relative gene expressions of UCP1, COX8b, PRDM16, Adcy5, Fabp4, HSL, Leptin, Adiponectin, and Resistin (*n* = 4). (C) PDGFRα+ progenitor cells from SAT transfected with miR‐379‐5p inhibitor or negative control (NC). Relative expression of SREBP1, C/EBPα, PPARγ, ACLY, FASN, DGAT1, FIT2, CIDEC, ATGL, and HSL (*n* = 3). (D) PDGFRα+ progenitor cells from SAT transfected with miR‐379‐5p mimics or NC. Relative expression of SREBP1, C/EBPα, PPARγ, ACLY, FASN, DGAT1, FIT2, CIDEC, ATGL, and HSL (*n* = 3). (E) miR‐379‐5p mimics or NC transfected into SI‐Exos from aged (20‐month‐old) mice, co‐cultured with PDGFRα+ progenitor cells. Gene expression of SREBP1, C/EBPα, PPARγ, ACLY, FASN, DGAT1, FIT2, CIDEC, ATGL, and HSL in miR‐379‐5p overexpression model (*n* = 3). Error bars represent ± SD. Comparisons analyzed by Student's t‐test. *p < 0.05, **p < 0.01, ***p < 0.001. 3m‐SI‐Exos, small intestinal epithelial exosomes from 3‐month‐old mice; NC, negative control.
**Figure S4:** scRNA‐seq analysis of SAT. scRNA‐seq of SAT isolated from aged mice receiving tail‐vein injections of PBS or 3m‐SI‐Exos (20 injections, every 3 days for 2 months) (*n* = 1). (A) Feature plot of PDGFRα expression across all cells. (B) Feature plot of PDGFRα in adipocytes. (C) Feature plot of PDGFRα in preadipocytes. (D) Violin plot illustrating PDGFRα expression across all cells. (E) DEGs between PDGFRα+ and PDGFRα− cells within preadipocytes. (F) GO term enrichment analysis of DEGs from PDGFRα+ preadipocytes. SAT, subcutaneous adipose tissue; 3m‐SI‐Exos, small intestinal epithelial exosomes from 3‐month‐old mice.
**Figure S5:** miR‐379‐5p directly targeted Usp34. (A, B) Small interfering RNAs (siRNAs) targeting Atm, together with negative control (NC), were transfected into PDGFRα+ progenitor cells. (A) Knockdown efficiency of Atm (*n* = 6). (B) Relative gene expression levels of PPARγ, ACLY, and CIDEC (*n* = 6). (C, D) siRNAs targeting Mtf1, together with NC, were transfected into PDGFRα+ progenitor cells. (C) Knockdown efficiency of Mtf1 (*n* = 6). (D) Relative gene expression levels of PPARγ, ACLY, and CIDEC (*n* = 6). (E–G) miR‐379‐5p, Usp34, and miR‐379‐5p inhibitor plus si‐Usp34 were individually introduced into PDGFRα+ progenitor cells. (E) Knockdown efficiency of Usp34. (F) Relative protein expression level of Usp34. (G) Relative gene expression levels of PPARγ, ACLY, and CIDEC. Error bars represent ± SD. Comparisons between two groups were analyzed by Student's t‐test. *p < 0.05, **p < 0.01, ***p < 0.001. NC, negative control; si‐Atm, small interfering RNA targeting Atm; si‐Mtf1, small interfering RNA targeting Mtf1; si‐Usp34, small interfering RNA targeting Usp34.
**Figure S6:** Wnt/β‐catenin pathway following Usp34 knockdown. PDGFRα+ progenitor cells were isolated from subcutaneous adipose tissue (SAT) by magnetic‐activated cell sorting (MACS) and transfected with Usp34 siRNA (si‐Usp34) or negative control (NC). (A) Usp34 protein expression at 72 h after transfection (*n* = 3). (B) Dual‐luciferase reporter assay showing β‐catenin/TCF transcriptional activity following Usp34 silencing. Cells transfected with NC or si‐Usp34 were subsequently co‐transfected with TOP‐Flash (containing eight TCF/LEF‐binding sites) or FOP‐Flash (mutant control) reporters. Luciferase activity was measured at 48 h after transfection and normalized to the Renilla/Firefly (RLUC/FLUC) ratio (*n* = 3). (C) Following transfection, cells underwent 6 days of adipogenic differentiation before BODIPY (green, lipid droplets) and DAPI (blue, nuclei) staining. Scale bar, 20 μm (*n* = 5). Error bars represent ± SD. Comparisons between two groups were analyzed by Student's t‐test. *p < 0.05, **p < 0.01, ***p < 0.001. si‐Usp34, small interfering RNA targeting Usp34; NC, negative control.
**Figure S7:** scRNA‐seq analysis of SAT. PBS or 3m‐SI‐Exos was administered to aged mice, and SAT was collected 2 months later for single‐cell RNA sequencing analysis. (A) Pseudotime trajectory of adipocytes and preadipocytes. SAT, subcutaneous adipose tissue. (B) GO enrichment analysis showing that DEGs between the exosome‐treated and control groups were enriched in muscle‐related pathways. SAT, subcutaneous adipose tissue; 3m‐SI‐Exos, small intestinal epithelial exosomes from 3‐month‐old mice.
**Figure S8:** scRNA‐seq analysis of macrophages in SAT and expression of inflammatory factors. PBS or 3m‐SI‐Exos was administered to aged mice, and SAT was collected 2 months later. (A) Relative mRNA expression levels of F4/80, IL‐1β, MCP1, NF‐κB, CXCL1, NLRP3, CCR2, and iNOS (*n* = 3). (B) UMAP visualization of macrophages from SAT scRNA‐seq data, colored by treatment group (3m‐SI‐Exos vs. PBS). (C) Dot plot displaying heterogeneous functional states of macrophage subpopulations. Error bars represent ± SD. Comparisons between two groups were analyzed by Student's t‐test. *p < 0.05, **p < 0.01, ***p < 0.001. SAT, subcutaneous adipose tissue; 3m‐SI‐Exos, small intestinal epithelial exosomes from 3‐month‐old mice.
**Figure S9:** scRNA‐seq analysis of SAT and VAT. scRNA‐seq was performed on SAT and VAT isolated from aged mice receiving tail‐vein injections of PBS or 3m‐SI‐Exos (20 injections, every 3 days for 2 months). (A) GO enrichment analysis of DEGs in SAT. (B) GO enrichment analysis of DEGs in VAT. SAT, subcutaneous adipose tissue; VAT, visceral adipose tissue; SI‐Exos, small intestinal epithelial exosomes.
**Table S1:** Primers used for real‐time PCR.
**Table S2:** Age‐related differentially expressed miRNAs.
**Table S3:** qPCR analysis of pre‐miR‐379 expression in SVF and SAT.

## Data Availability

Additional data will be shared upon request.
