# Supplementary material for: From Gut to Fat: Intestinal Epithelial Exosomes Target PDGFRα + Progenitors to Promote Lipogenesis and Counteract Subcutaneous Adipose Tissue Atrophy in Aging
Source: Aging Cell. 2026 Jul 12;25(7):e70625. doi: 10.1111/acel.70625 (PMC13357384; doi:10.1111/acel.70625)
Supplement: Supplementary file 1 — Figure S1: Comprehensive characterization of exosome preparations. (A) Transmission electron microscopy showing typical cup‐shaped morphology with a diameter of approximately 100 nm, consistent with exosome ultrastructure. (B) Nanoparticle tracking analysis revealing a size distribution peak between 100 and 120 nm. (C) Western blot analysis demonstrating enrichment of exosome markers (GPA33, TSG101, and CD63) and the absence of cellular contamination marker (Calnexin). Scale bar, 100 nm. Exos, exosomes. Figure S2: Effects of fecal microbiota transplantation on adipose tissue. Eight‐week‐old germ‐free C57BL/6J mice received FMT from young (3‐month‐old) or aged (20‐month‐old) donors mice, or PBS, twice (200 mg/dose) in 1 week. Subcutaneous adipose tissue was analyzed after one additional week. (A) Representative images and body weights of mice after transplantation. (B) Adipose tissue morphology and weights of SAT and VAT (n = 3). (C) H&E staining of adipocytes from SAT and VAT (n = 3). Scale bar, 50 μm. Error bars represent ± SD. Comparison between two groups was performed by Student's t‐test. *p < 0.05, **p < 0.01, ***p < 0.001. SI‐Exos, small intestinal epithelial exosomes; SAT, subcutaneous adipose tissue; VAT, visceral adipose tissue. Figure S3: Relative gene expression of lipogenesis‐related genes. (A) 20‐month‐old mice received tail‐vein injections of SI‐Exos derived from 3‐month‐old mice (100 μg per injection, every 3 days for 2 months, total 20 injections). Control mice received an equal volume of PBS. Gene expression related to adipocyte differentiation, triglyceride and fatty acid synthesis, lipid droplet formation, and lipolysis (n = 3). (B) PDGFRα+ and PDGFRα− progenitor cells were isolated using magnetic beads and induced to undergo adipogenesis for 6 days. Relative gene expressions of UCP1, COX8b, PRDM16, Adcy5, Fabp4, HSL, Leptin, Adiponectin, and Resistin (n = 4). (C) PDGFRα+ progenitor cells from SAT transfected with miR‐379‐5p inhibitor or negative [file ACEL-25-e70625-s001.zip › acel70625-sup-0001-FigureS1-S9-TableS1-S3@Revised Supplementary Material -6.9.docx]

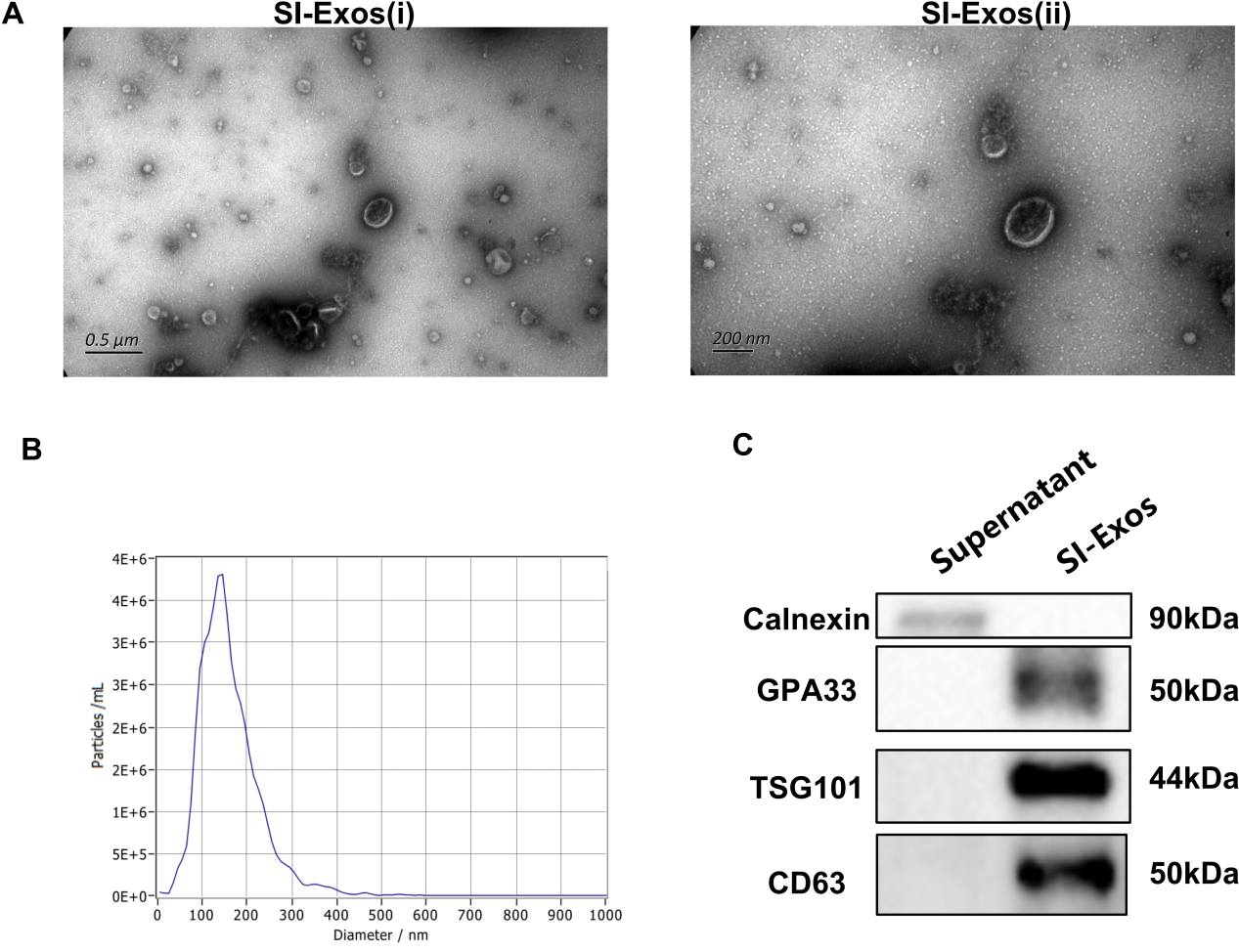


Supplementary Figure 1 Comprehensive characterization of exosome preparations

(A) Transmission electron microscopy showing typical cup-shaped morphology with a diameter of approximately 100 nm, consistent with exosome ultrastructure. (B) Nanoparticle tracking analysis revealing a size distribution peak between 100-120 nm. (C) Western blot analysis demonstrating enrichment of exosome markers (GPA33, TSG101, and CD63) and the absence of cellular contamination marker (Calnexin). Scale bar, 100 nm. Exos, exosomes.


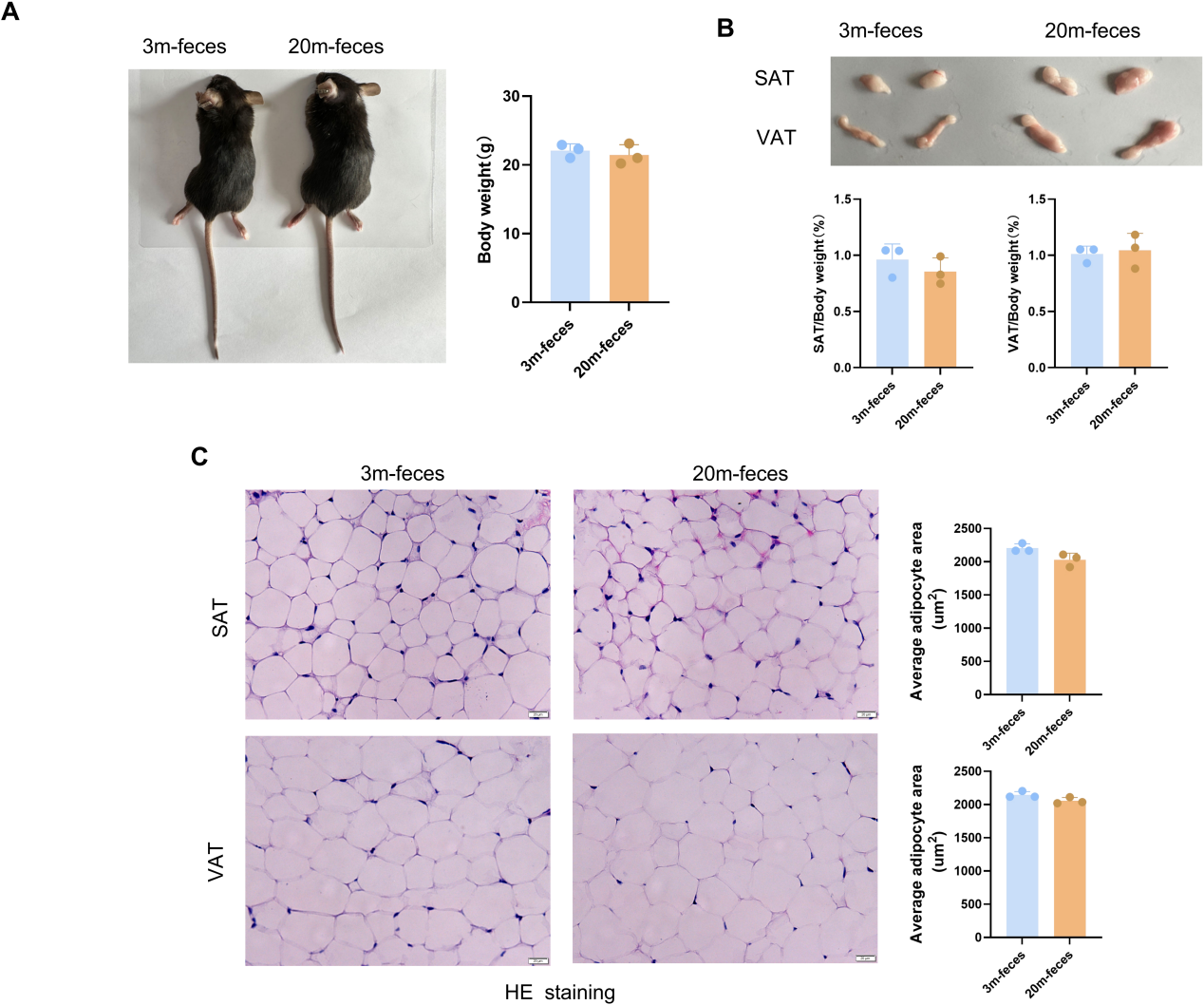


Supplementary Figure 2 Effects of fecal microbiota transplantation on adipose tissue

Eight-week-old germ-free C57BL/6J mice received FMT from young (3-month-old) or aged (20-month-old) donors mice, or PBS, twice (200 mg/dose) in one week. Subcutaneous adipose tissue was analyzed after one additional week. (A) Representative images and body weights of mice after transplantation. (B) Adipose tissue morphology and weights of SAT and VAT (n = 3). (C) H&E staining of adipocytes from SAT and VAT (n = 3). Scale bar, 50 μm. Error bars represent ± SD. Comparison between two groups was performed by Student’s t-test. **p*<0.05, ***p*<0.01, ****p*<0.001.

SI-Exos, small intestinal epithelial exosomes; SAT, subcutaneous adipose tissue; VAT, visceral adipose tissue.


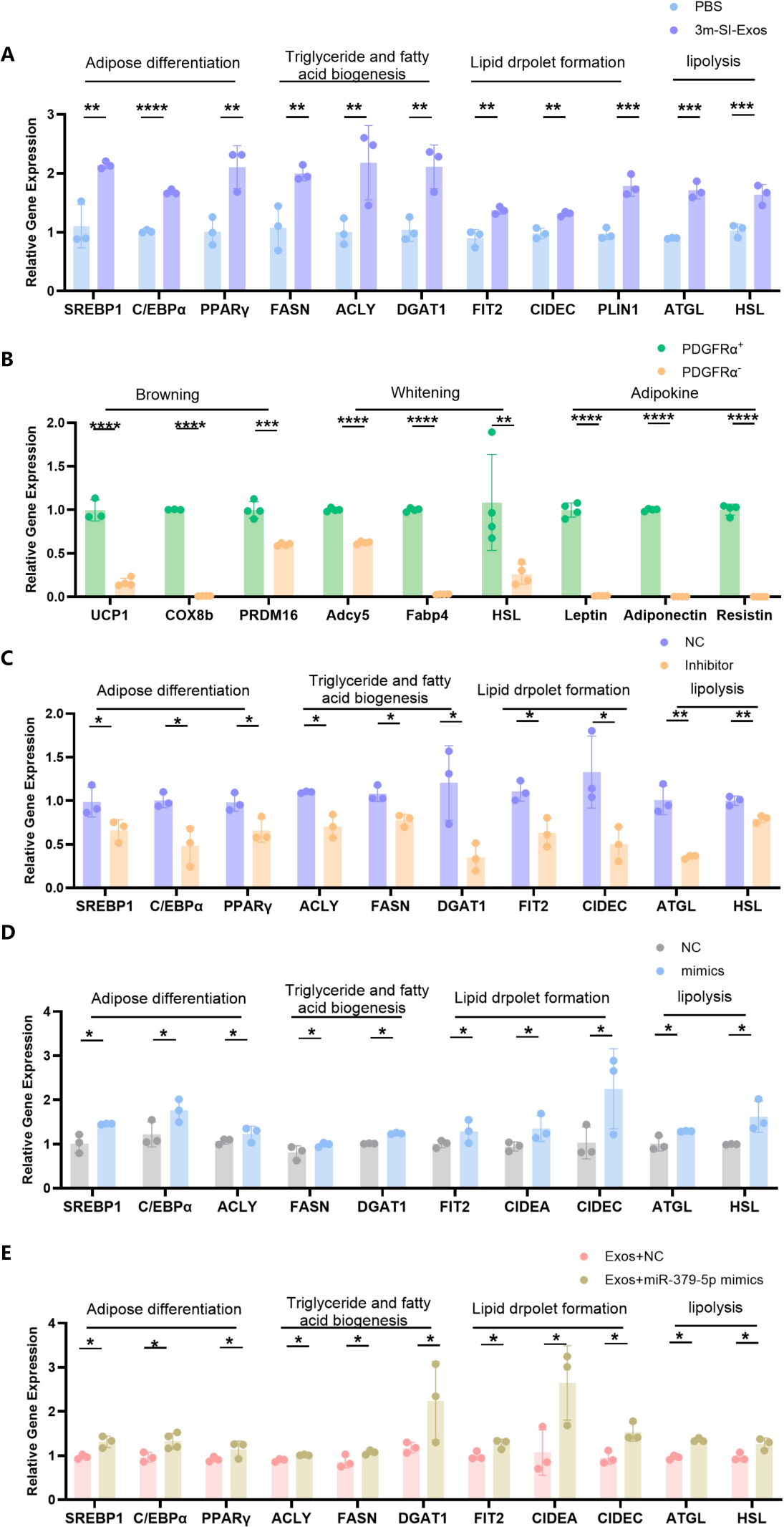


Supplementary Figure 3 Relative gene expression of lipogenesis-related genes

(A) 20-month-old mice received tail-vein injections of SI-Exos derived from 3-month-old mice (100 μg per injection, every 3 days for 2 months, total 20 injections). Control mice received an equal volume of PBS. Gene expression related to adipocyte differentiation, triglyceride and fatty acid synthesis, lipid droplet formation, and lipolysis (n = 3). (B) PDGFRα^+^ and PDGFRα^-^ progenitor cells were isolated using magnetic beads and induced to undergo adipogenesis for 6 days. Relative gene expressions of UCP1, COX8b, PRDM16, Adcy5, Fabp4, HSL, Leptin, Adiponectin, and Resistin (n = 4). (C) PDGFRα^+^ progenitor cells from SAT transfected with miR-379-5p inhibitor or negative control (NC). Relative expression of SREBP1, C/EBPα, PPARγ, ACLY, FASN, DGAT1, FIT2, CIDEC, ATGL, and HSL (n = 3). (D) PDGFRα^+^ progenitor cells from SAT transfected with miR-379-5p mimics or NC. Relative expression of SREBP1, C/EBPα, PPARγ, ACLY, FASN, DGAT1, FIT2, CIDEC, ATGL, and HSL (n = 3). (E) miR-379-5p mimics or NC transfected into SI-Exos from aged (20-month-old) mice, co-cultured with PDGFRα^+^ progenitor cells. Gene expression of SREBP1, C/EBPα, PPARγ, ACLY, FASN, DGAT1, FIT2, CIDEC, ATGL, and HSL in miR-379-5p overexpression model (n = 3). Error bars represent ± SD. Comparisons analyzed by Student’s t-test. **p*<0.05, ***p*<0.01, ****p*<0.001.

3m-SI-Exos, small intestinal epithelial exosomes from 3-month-old mice; NC, negative control.


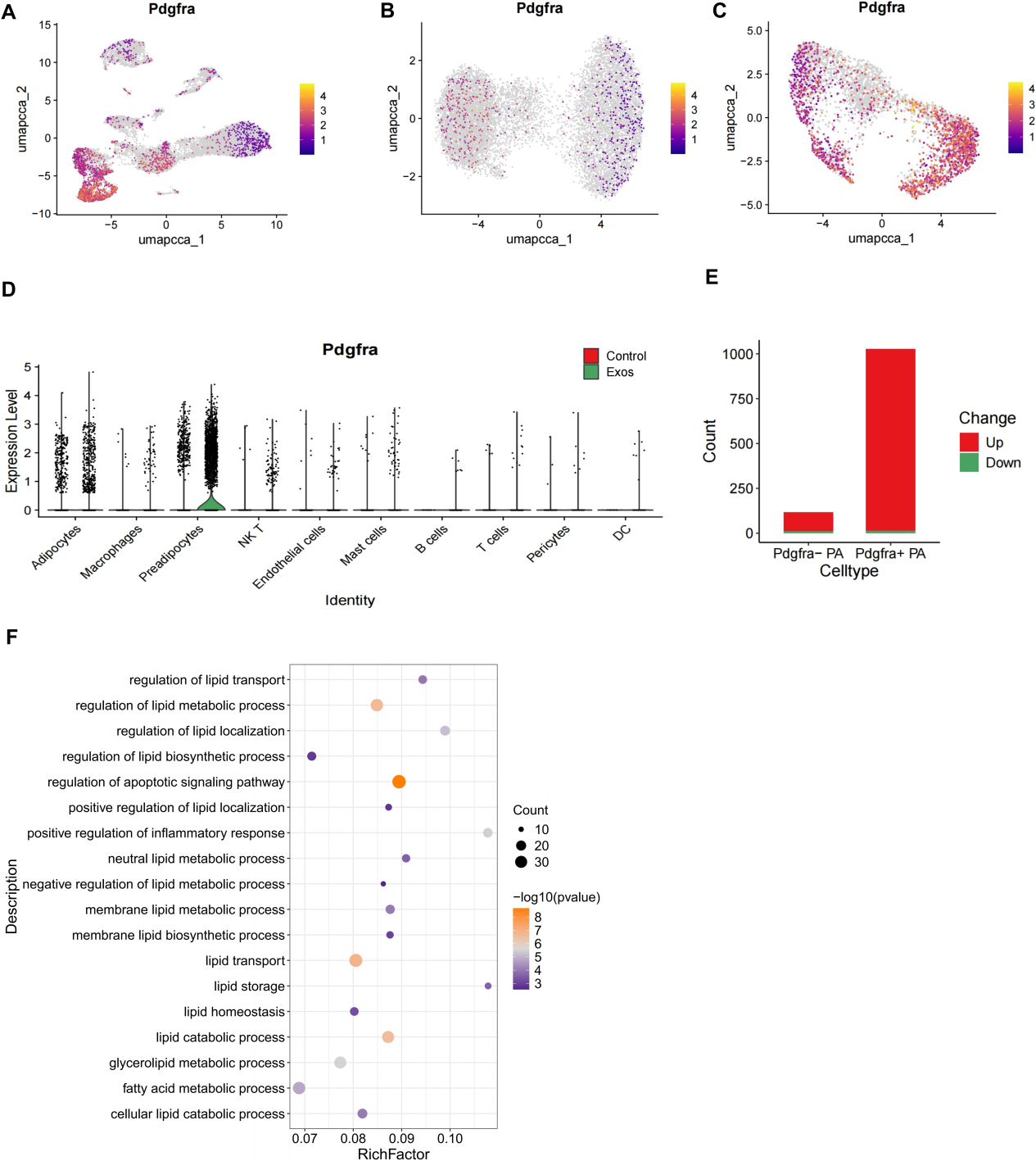


Supplementary Figure 4 scRNA-seq analysis of SAT

scRNA-seq of SAT isolated from aged mice receiving tail-vein injections of PBS or 3m-SI-Exos (20 injections, every 3 days for 2 months) (n = 1). (A) Feature plot of PDGFRα expression across all cells. (B) Feature plot of PDGFRα in adipocytes. (C) Feature plot of PDGFRα in preadipocytes. (D) Violin plot illustrating PDGFRα expression across all cells. (E) DEGs between PDGFRα^+^ and PDGFRα^-^ cells within preadipocytes. (F) GO term enrichment analysis of DEGs from PDGFRα^+^ preadipocytes.

SAT, subcutaneous adipose tissue; 3m-SI-Exos, small intestinal epithelial exosomes from 3-month-old mice.


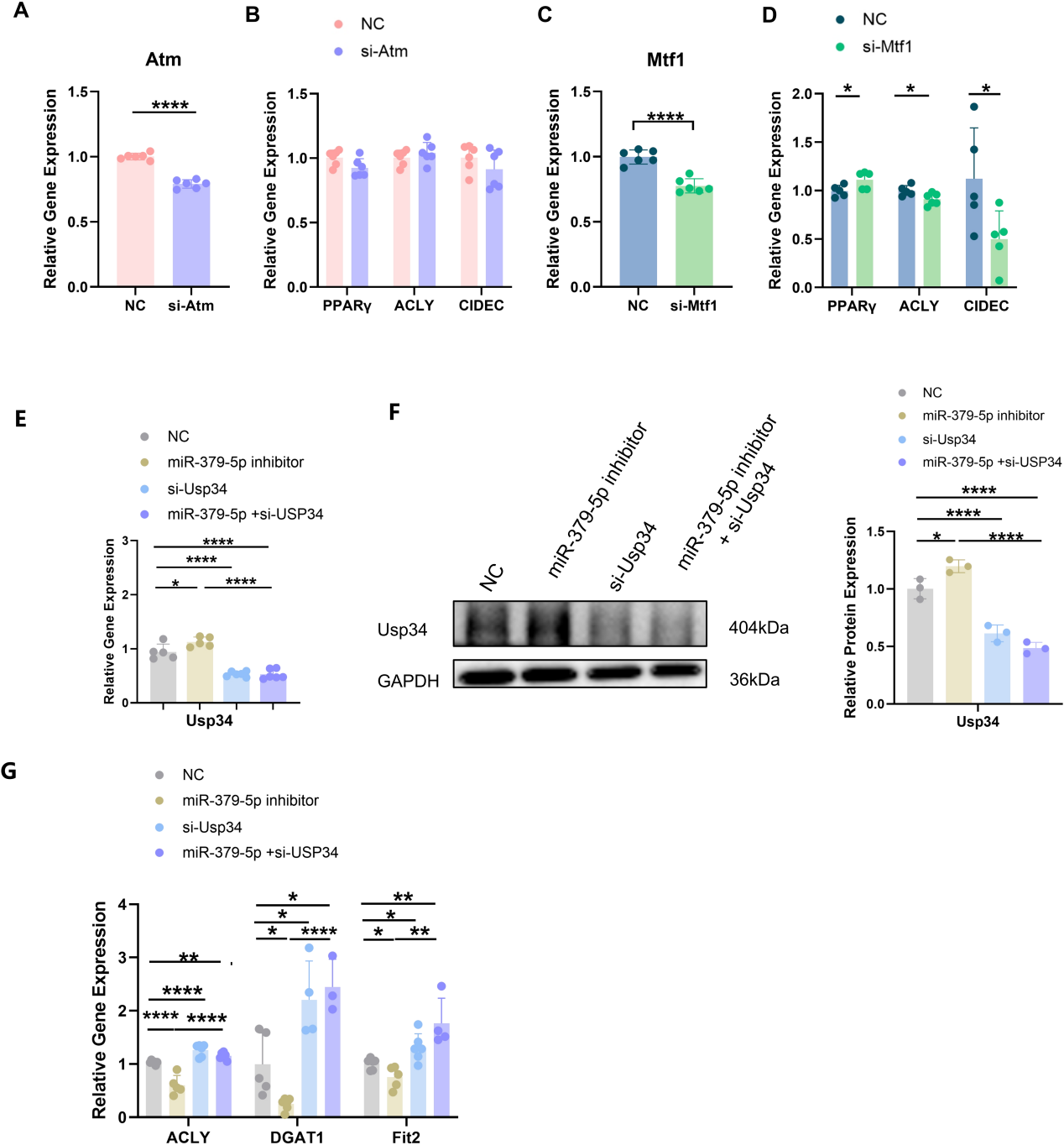


Supplementary Figure 5 miR-379-5p directly targeted Usp34.

(A-B) Small interfering RNAs (siRNAs) targeting Atm, together with negative control (NC), were transfected into PDGFRα^+^ progenitor cells. (A) Knockdown efficiency of Atm (n = 6). (B) Relative gene expression levels of PPARγ, ACLY, and CIDEC (n = 6). (C-D) siRNAs targeting Mtf1, together with NC, were transfected into PDGFRα^+^ progenitor cells. (C) Knockdown efficiency of Mtf1 (n = 6). (D) Relative gene expression levels of PPARγ, ACLY, and CIDEC (n = 6). (E-G) miR-379-5p, Usp34, and miR-379-5p inhibitor plus si-Usp34 were individually introduced into PDGFRα^+^ progenitor cells. (E) Knockdown efficiency of Usp34. (F) Relative protein expression level of Usp34. (G) Relative gene expression levels of PPARγ, ACLY, and CIDEC. Error bars represent ± SD. Comparisons between two groups were analyzed by Student’s t-test. **p*<0.05, ***p*<0.01, ****p*<0.001.

NC, negative control; si-Atm, small interfering RNA targeting Atm; si-Mtf1, small interfering RNA targeting Mtf1; si-Usp34, small interfering RNA targeting Usp34.


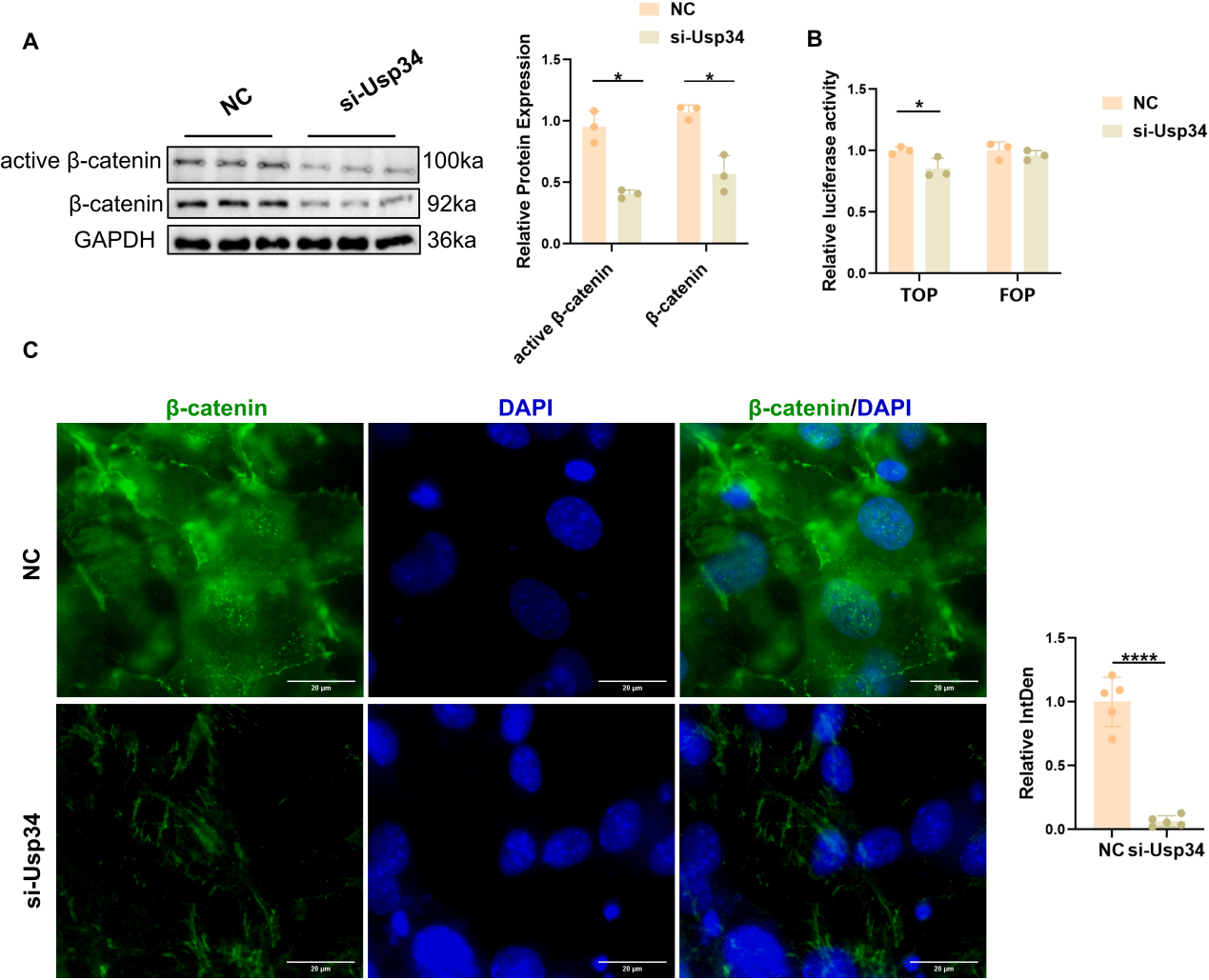


Supplementary Figure 6 Wnt/β-catenin pathway following Usp34 knockdown

PDGFRα^+^ progenitor cells were isolated from subcutaneous adipose tissue (SAT) by magnetic-activated cell sorting (MACS) and transfected with Usp34 siRNA (si-Usp34) or negative control (NC). (A) Usp34 protein expression at 72 h after transfection (n = 3). (B) Dual-luciferase reporter assay showing β-catenin/TCF transcriptional activity following Usp34 silencing. Cells transfected with NC or si-Usp34 were subsequently co-transfected with TOP-Flash (containing eight TCF/LEF-binding sites) or FOP-Flash (mutant control) reporters. Luciferase activity was measured at 48 h after transfection and normalized to the Renilla/Firefly (RLUC/FLUC) ratio (n = 3). (C) Following transfection, cells underwent 6 days of adipogenic differentiation before BODIPY (green, lipid droplets) and DAPI (blue, nuclei) staining. Scale bar, 20 μm (n = 5). Error bars represent ± SD. Comparisons between two groups were analyzed by Student’s t-test. **p*<0.05, ***p*<0.01, ****p*<0.001. si-Usp34, small interfering RNA targeting Usp34; NC, negative control.


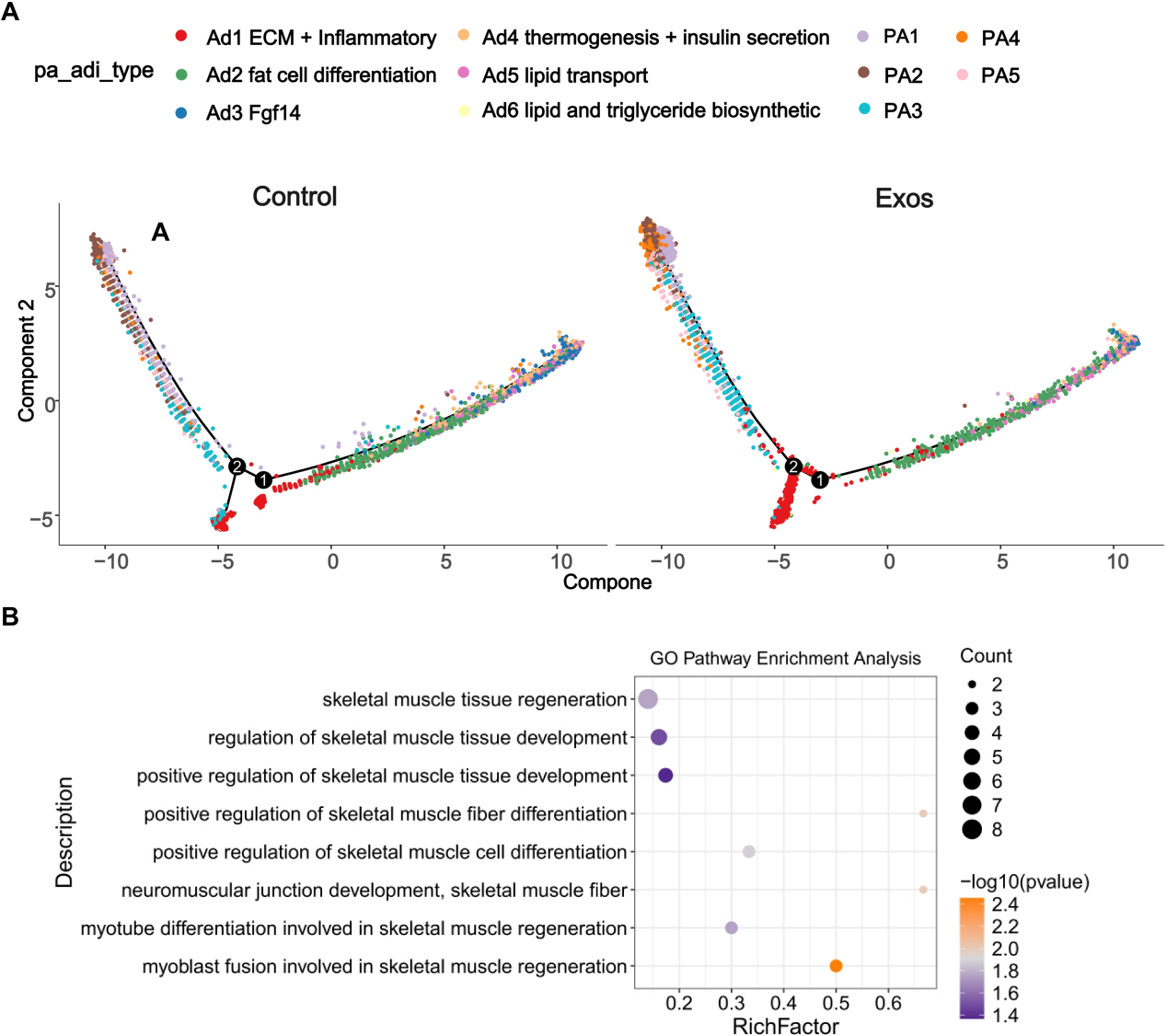


Supplementary Figure 7 scRNA-seq analysis of SAT

PBS or 3m-SI-Exos was administered to aged mice, and SAT was collected 2 months later for single-cell RNA sequencing analysis. (A) Pseudotime trajectory of adipocytes and preadipocytes. SAT, subcutaneous adipose tissue. (B) GO enrichment analysis showing that DEGs between the exosome-treated and control groups were enriched in muscle-related pathways. SAT, subcutaneous adipose tissue; 3m-SI-Exos, small intestinal epithelial exosomes from 3-month-old mice.


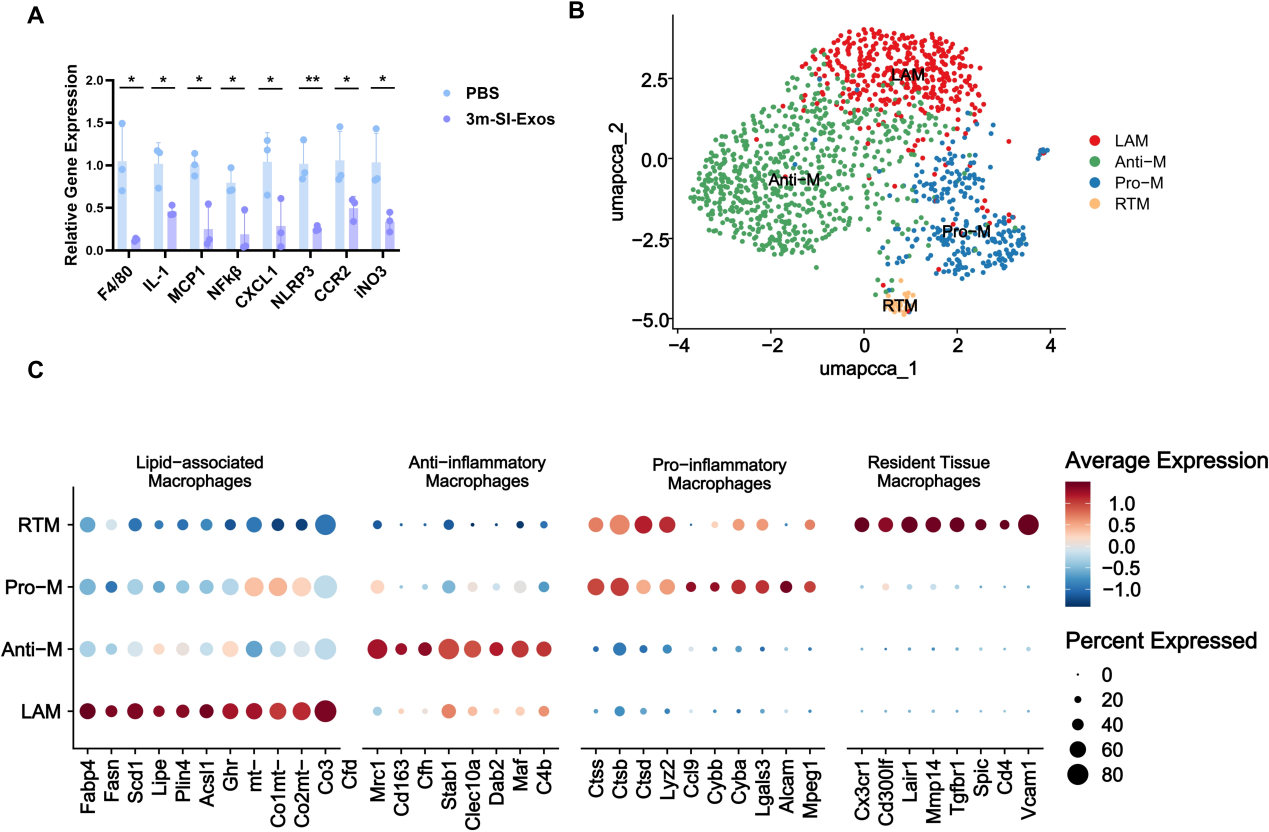


Supplementary Figure 8 scRNA-seq analysis of macrophages in SAT and expression of inflammatory factors

PBS or 3m-SI-Exos was administered to aged mice, and SAT was collected 2 months later. (A) Relative mRNA expression levels of F4/80, IL-1β, MCP1, NF-κB, CXCL1, NLRP3, CCR2, and iNOS (n = 3). (B) UMAP visualization of macrophages from SAT scRNA-seq data, colored by treatment group (3m-SI-Exos vs. PBS). (C) Dot plot displaying heterogeneous functional states of macrophage subpopulations. Error bars represent ± SD. Comparisons between two groups were analyzed by Student’s t-test. **p*<0.05, ***p*<0.01, ****p*<0.001. SAT, subcutaneous adipose tissue; 3m-SI-Exos, small intestinal epithelial exosomes from 3-month-old mice.


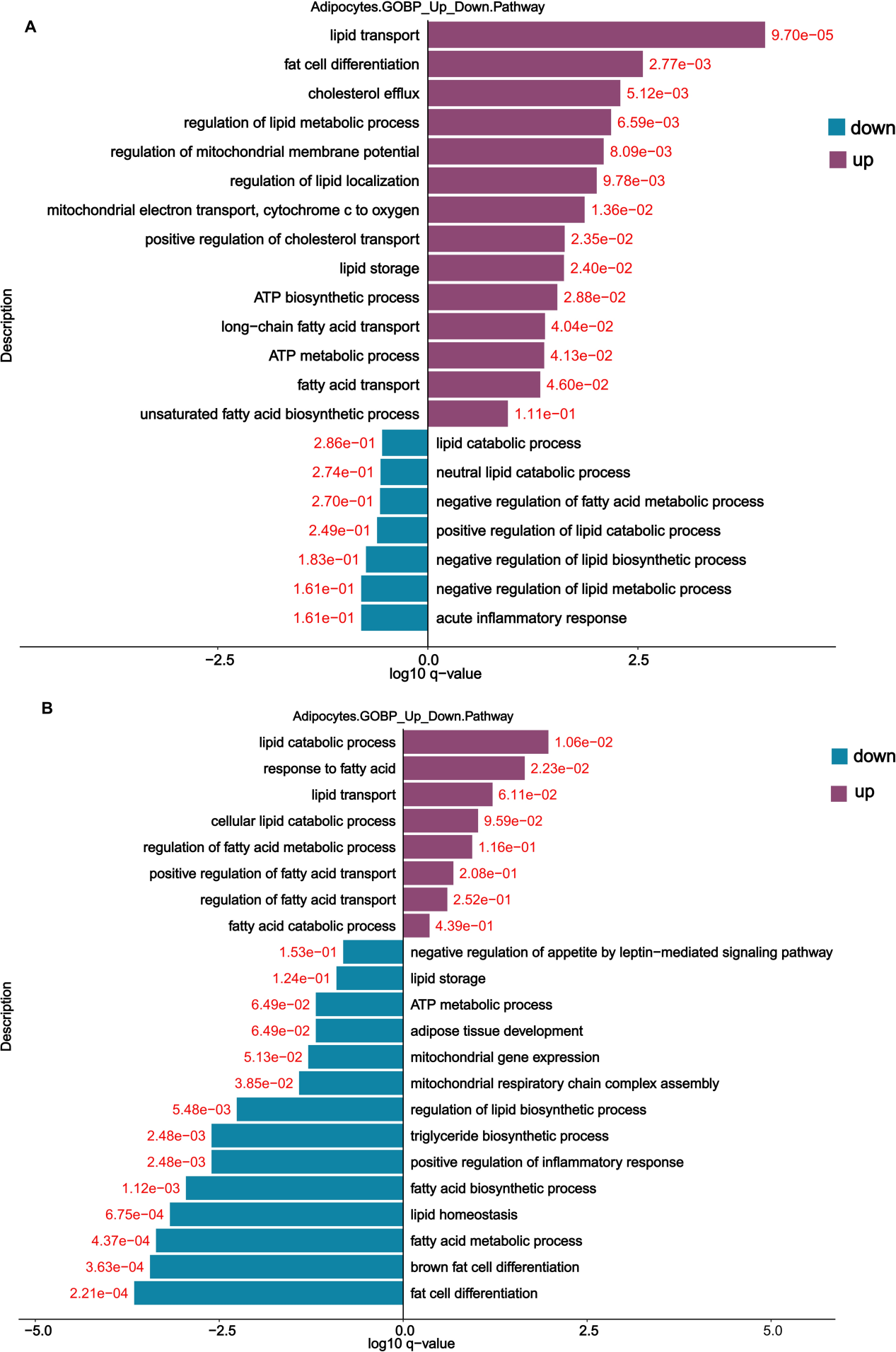


Supplementary Figure 9 scRNA-seq analysis of SAT and VAT

scRNA-seq was performed on SAT and VAT isolated from aged mice receiving tail-vein injections of PBS or 3m-SI-Exos (20 injections, every 3 days for 2 months). (A) GO enrichment analysis of DEGs in SAT. (B) GO enrichment analysis of DEGs in VAT.

SAT, subcutaneous adipose tissue; VAT, visceral adipose tissue; SI-Exos, small intestinal epithelial exosomes.

Supplementary Table 1 Primers used for real-time PCR

| Gene | Prime | |
| --- | --- | --- |
| β-actin | forward | 5’-GGGACCTGTGAGTGCTTCC-3’ |
|  | reverse | 5’-GTATTGAAGAGCCGGGATCTTTT-3’ |
| UCP1 | forward | 5’-AGGGTTTGTGGCTTCTTTTC-3’ |
|  | reverse | 5’-TGGTTGGTTTTATTCGTGGT-3’ |
| Cox8b | forward | 5’-GGAGTGCGACCCCGAGAAT-3’ |
|  | reverse | 5’-CGGCGGAAGTGGGAGTTTT-3’ |
| PRDM16 | forward | 5’-CAGCACGGTGAAGCCATTC-3’ |
|  | reverse | 5’-GCG TGCATCCGCTTGTG-3’ |
| Adcy5 | forward | 5’-CTTGGGGAGAAGCCGATTCC-3’ |
|  | reverse | 5’-ACCGCTTAGTGGAGGGTCT-3’ |
| Adiponectin | forward | 5’-AGGTCTTCTTGGTCATAAGGGTG-3’ |
|  | reverse | 5’-TTGCCAGTGCTGCCGTCATA-3’ |
| HSL | forward | 5’-CCACACGGGAAGAAGACTAGC-3’ |
|  | reverse | 5’-CAGTTGGCCTAGGGTTGGTT-3’ |
| Resistin | forward | 5’-CTTGCCAATCGAGATGACTGT-3’ |
|  | reverse | 5’-GTCTGCCTGAAGCCGTGATAC-3’ |
| PPARγ | forward | 5’-GACCACTCGCATTCCTTT-3’ |
|  | reverse | 5’-CCACAGACTCGGCACTCA-3’ |
| C/EBPα | forward | 5’-GTCACTGGTCAACTCCAGCAC-3’ |
|  | reverse | 5’-CAAGAACAGCAACGAGTACCG-3’ |
| SREBP1 | forward | 5′-TGACCCGGCTATTCCGTGA-3′ |
|  | reverse | 5′-CTGGGCTGAGCAATACAGTTC-3′ |
| PLIN1 | forward | 5’-GGGACCTGTGAGTGCTTCC-3’ |
|  | reverse | 5’-GTATTGAAGAGCCGGGATCTTTT-3’ |
| ACLY | forward | 5’-TTCCTCCTTAATGCCAGCGG-3’ |
|  | reverse | 5’-TGCAGGGATCTTGGACTTGG-3’ |
| FASN | forward | 5’-TGCCTTCGGTTCAGTCTCTT-3’ |
|  | reverse | 5’-CACCCTCCAAGGAGTCTCAC-3’ |
| DGAT1 | forward | 5’-CGTCGCAGAGGTAGTCGTG-3’ |
|  | reverse | 5’-CTACCACCCTGGACGGAAAC-3’ |
| ATGL | forward | 5’-TTCGCAATCTCTACCGCCTC-3’ |
|  | reverse | 5’-AGCAAAGGGTTGGGTTGGTT-3’ |
| FIT2 | forward | 5’-TCCTGCCTTTCATTGCCCTT-3’ |
|  | reverse | 5’-AGTGGCCCGAGATGTCAAAG-3’ |
| CIDEC | forward | 5’-GTGTCCACTTGTGCCGTCT-3’ |
|  | reverse | 5’-TGCTCGCTTGGTTGTCTTG-3’ |
| Klf12 | forward | 5’-CAGCGCCCTTGAGAACAGAAT-3’ |
|  | reverse | 5’-GTGGACGTTTGGAGACCCTTG-3’ |
| C-myc | forward | 5’-GGACCCATCTACAGAGGCTG-3’ |
|  | reverse | 5’-ATCACAATGGTGGAGGGTGC-3’ |
| Cyclin D1 | forward | 5’-CATTGTCCCCCGAGATAGCC-3’ |
|  | reverse | 5’-CCATCCTGTCTGGTGGAACC-3’ |
| Fbn2 | forward | 5’-CTCCACCAAAGACGCTCTGG-3’ |
|  | reverse | 5’-CCCTCGTCCCGATACTCAGG-3’ |
| Usp34 | forward | 5’-GATATTGGTGGTCGTTCATGTGT-3’ |
|  | reverse | 5’-TTGGCAAATTCGTAAAGGAAAGC-3’ |
| Atm | forward | 5’-TTGGCAAATTCGTAAAGGAAAGC-3’ |
|  | reverse | 5’-CAGCACACTTCTTTCCACCAC-3’ |
| Mtf1 | forward | 5’-TTTCGTCCTCGAACCAGCTC-3’ |
|  | reverse | 5’-TACACCAGGGAATGCACGTC-3’ |
| Ctnnb1 | forward | 5’-GTTCGCCCCCTTTAATAGTGC-3’ |
|  | reverse | 5’-TGAACTCCAACGTCAAGCGG-3’ |

Supplementary Table 2 Age-related differentially expressed miRNAs

| 102 age-downregulated miRNAs | | | | |
| --- | --- | --- | --- | --- |
| mmu-miR-200b-3p | mmu-miR-1949 | mmu-miR-429-3p | mmu-miR-143-3p | mmu-miR-378a-3p |
| mmu-miR-7b-5p | mmu-miR-20b-5p | mmu-miR-200a-3p | mmu-let-7c-5p | mmu-miR-16-5p |
| mmu-miR-203-3p | mmu-miR-369-5p | mmu-miR-1a-3p | mmu-let-7b-5p | mmu-miR-15b-5p |
| mmu-miR-802-5p | mmu-miR-24-2-5p | mmu-let-7i-5p | mmu-miR-183-5p | mmu-miR-22-3p |
| mmu-miR-203-5p | mmu-miR-467a-5p | mmu-miR-154-5p | mmu-miR-30d-5p | mmu-let-7e-5p |
| mmu-miR-28a-3p | mmu-miR-196a-1-3p | mmu-let-7d-5p | mmu-let-7g-5p | mmu-miR-145a-5p |
| mmu-miR-1983 | mmu-miR-296-3p | mmu-miR-127-3p | mmu-miR-411-5p | mmu-miR-30b-5p |
| novel-mmu-miR380-5p | mmu-miR-802-3p | mmu-miR-139-5p | mmu-miR-26b-5p | mmu-miR-24-3p |
| mmu-miR-378b | mmu-miR-192-3p | novel-mmu-miR220-5p | mmu-miR-10a-5p | novel-mmu-miR433-5p |
| novel-mmu-miR101-5p | mmu-miR-20a-5p | mmu-miR-192-5p | mmu-miR-26a-5p | mmu-miR-150-5p |
| novel-mmu-miR72-3p | mmu-miR-98-5p | mmu-miR-7a-5p | mmu-miR-218-5p | mmu-miR-99b-5p |
| novel-mmu-miR143-3p | mmu-miR-3470a | mmu-miR-9b-3p | mmu-miR-872-5p | mmu-miR-126a-5p |
| mmu-miR-341-3p | mmu-miR-7a-1-3p | mmu-miR-182-5p | mmu-miR-146a-5p | mmu-miR-31-5p |
| mmu-miR-181d-5p | mmu-miR-374b-5p | mmu-miR-375-3p | mmu-miR-194-5p | mmu-miR-195a-5p |
| novel-mmu-miR308-5p | mmu-miR-93-5p | mmu-miR-27b-3p | mmu-miR-191-5p |  |
| novel-mmu-miR75-5p | mmu-miR-379-5p | mmu-let-7a-5p | mmu-miR-1981-5p |  |
| mmu-miR-3068-5p | mmu-miR-25-3p | novel-mmu-miR24-5p | mmu-miR-652-3p |  |
| novel-mmu-miR361-3p | mmu-miR-30c-5p | mmu-miR-3470b | mmu-miR-27a-3p |  |
| mmu-miR-203b-5p | mmu-miR-1839-5p | mmu-miR-191-3p | mmu-miR-196a-5p |  |
| mmu-miR-328-5p | mmu-miR-200c-3p | mmu-let-7d-3p | mmu-miR-23b-3p |  |
| mmu-miR-872-3p | mmu-let-7f-5p | mmu-miR-378c | mmu-miR-34a-5p |  |
| mmu-miR-200b-5p | mmu-miR-151-5p | mmu-miR-200a-5p | mmu-miR-181a-5p |  |

High-throughput miRNA sequencing was performed on SI-Exos isolated from 3-, 8-, 15-, and 18-month-old mice.

SI-Exos, small intestinal epithelial exosomes.

Supplementary Table 3 qPCR analysis of pre-miR-379 expression in SVF and SAT

|  | Group | Ct(U6) | Ct(pre-miR-379) |
| --- | --- | --- | --- |
| SVF | 3m SVF 1 | 12.69 | 35 |
|  | 3m SVF 2 | 12.79 | 35 |
|  | 3m SVF 3 | 13.55 | - |
|  | 3m SVF 4 | 13.26 | 35 |
|  | 20m SVF 1 | 15.2 | 35 |
|  | 20m SVF 2 | 15 | 35 |
|  | 20m SVF 3 | 15.47 | 35 |
|  | 20m SVF 4 | 15.65 | - |
|  |  |  |  |
| SAT | 3m SAT 1 | 11.72 | 35 |
|  | 3m SAT 2 | 12.29 | 35 |
|  | 3m SAT 3 | 10.43 | 35 |
|  | 3m SAT 4 | 14.01 | - |
|  | 3m SAT 5 | 13.24 | 35 |
|  | 3m SAT 6 | 12.75 | 35 |
|  | 20m SAT 1 | 16.58 | - |
|  | 20m SAT 2 | 17.87 | - |
|  | 20m SAT 3 | 19.26 | - |
|  | 20m SAT 4 | 18.48 | - |
|  | 20m SAT 5 | 18.46 | - |
|  | 20m SAT 6 | 17.81 | - |

Total RNA was extracted from SAT and SVF, followed by stem-loop RT-qPCR using pre-miR-379-specific primers. U6 snRNA served as the internal control. Pre-miR-379 was undetectable (Ct > 35 or undetermined after 40 amplification cycles) in both SVF (n = 4) and SAT (n = 6).

SAT, subcutaneous adipose tissue; SVF, stromal vascular fraction.
