# Supplementary material for: From Gut to Fat: Intestinal Epithelial Exosomes Target PDGFRα + Progenitors to Promote Lipogenesis and Counteract Subcutaneous Adipose Tissue Atrophy in Aging
Source: Aging Cell. 2026 Jul 12;25(7):e70625. doi: 10.1111/acel.70625 (PMC13357384; doi:10.1111/acel.70625)
Supplement: Supplementary file 1 — Figure S1: Comprehensive characterization of exosome preparations. (A) Transmission electron microscopy showing typical cup‐shaped morphology with a diameter of approximately 100 nm, consistent with exosome ultrastructure. (B) Nanoparticle tracking analysis revealing a size distribution peak between 100 and 120 nm. (C) Western blot analysis demonstrating enrichment of exosome markers (GPA33, TSG101, and CD63) and the absence of cellular contamination marker (Calnexin). Scale bar, 100 nm. Exos, exosomes. Figure S2: Effects of fecal microbiota transplantation on adipose tissue. Eight‐week‐old germ‐free C57BL/6J mice received FMT from young (3‐month‐old) or aged (20‐month‐old) donors mice, or PBS, twice (200 mg/dose) in 1 week. Subcutaneous adipose tissue was analyzed after one additional week. (A) Representative images and body weights of mice after transplantation. (B) Adipose tissue morphology and weights of SAT and VAT (n = 3). (C) H&E staining of adipocytes from SAT and VAT (n = 3). Scale bar, 50 μm. Error bars represent ± SD. Comparison between two groups was performed by Student's t‐test. *p < 0.05, **p < 0.01, ***p < 0.001. SI‐Exos, small intestinal epithelial exosomes; SAT, subcutaneous adipose tissue; VAT, visceral adipose tissue. Figure S3: Relative gene expression of lipogenesis‐related genes. (A) 20‐month‐old mice received tail‐vein injections of SI‐Exos derived from 3‐month‐old mice (100 μg per injection, every 3 days for 2 months, total 20 injections). Control mice received an equal volume of PBS. Gene expression related to adipocyte differentiation, triglyceride and fatty acid synthesis, lipid droplet formation, and lipolysis (n = 3). (B) PDGFRα+ and PDGFRα− progenitor cells were isolated using magnetic beads and induced to undergo adipogenesis for 6 days. Relative gene expressions of UCP1, COX8b, PRDM16, Adcy5, Fabp4, HSL, Leptin, Adiponectin, and Resistin (n = 4). (C) PDGFRα+ progenitor cells from SAT transfected with miR‐379‐5p inhibitor or negative [file ACEL-25-e70625-s001.zip › acel70625-sup-0003-FigureS1-S4-TableS1@Supplementary Material.docx]

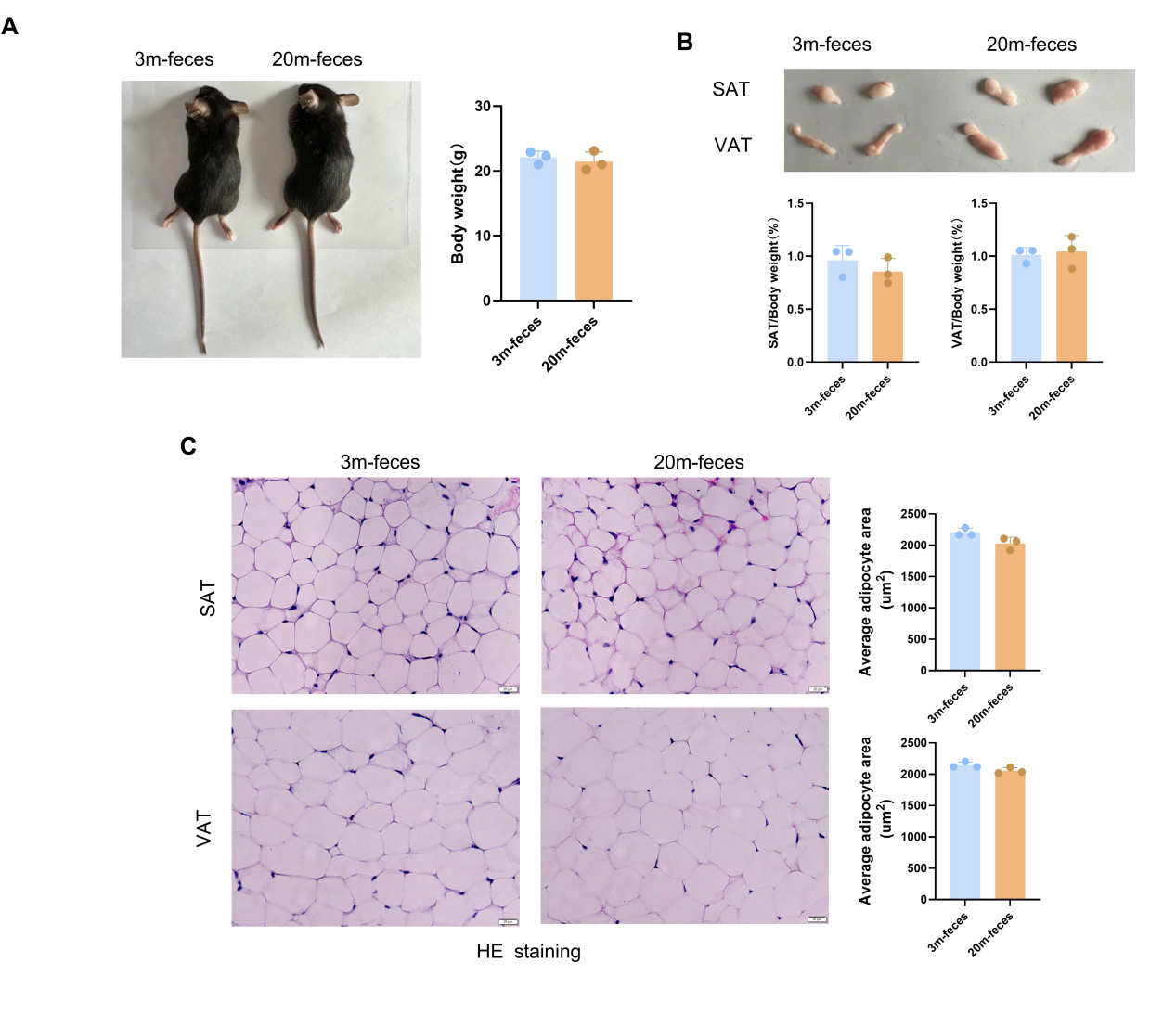


Supplementary Figure 1 Gut microbiota from young (3 months) and aged (20 months) mice were transplanted into germ-free (8 weeks) separately. (A)Representative images of mice after transplantation and body weight. (B)Adipose morphology and weights of SAT and VAT in Mice. (C)Representative mages of HE staining in SAT and VAT. Scale bar, 50 μm. **p*<0.05, ***p*<0.01, ****p*<0.001.

SI-Exos: small intestinal epithelial exosomes; SAT: subcutaneous adipose tissue; VAT, visceral adipose tissue.


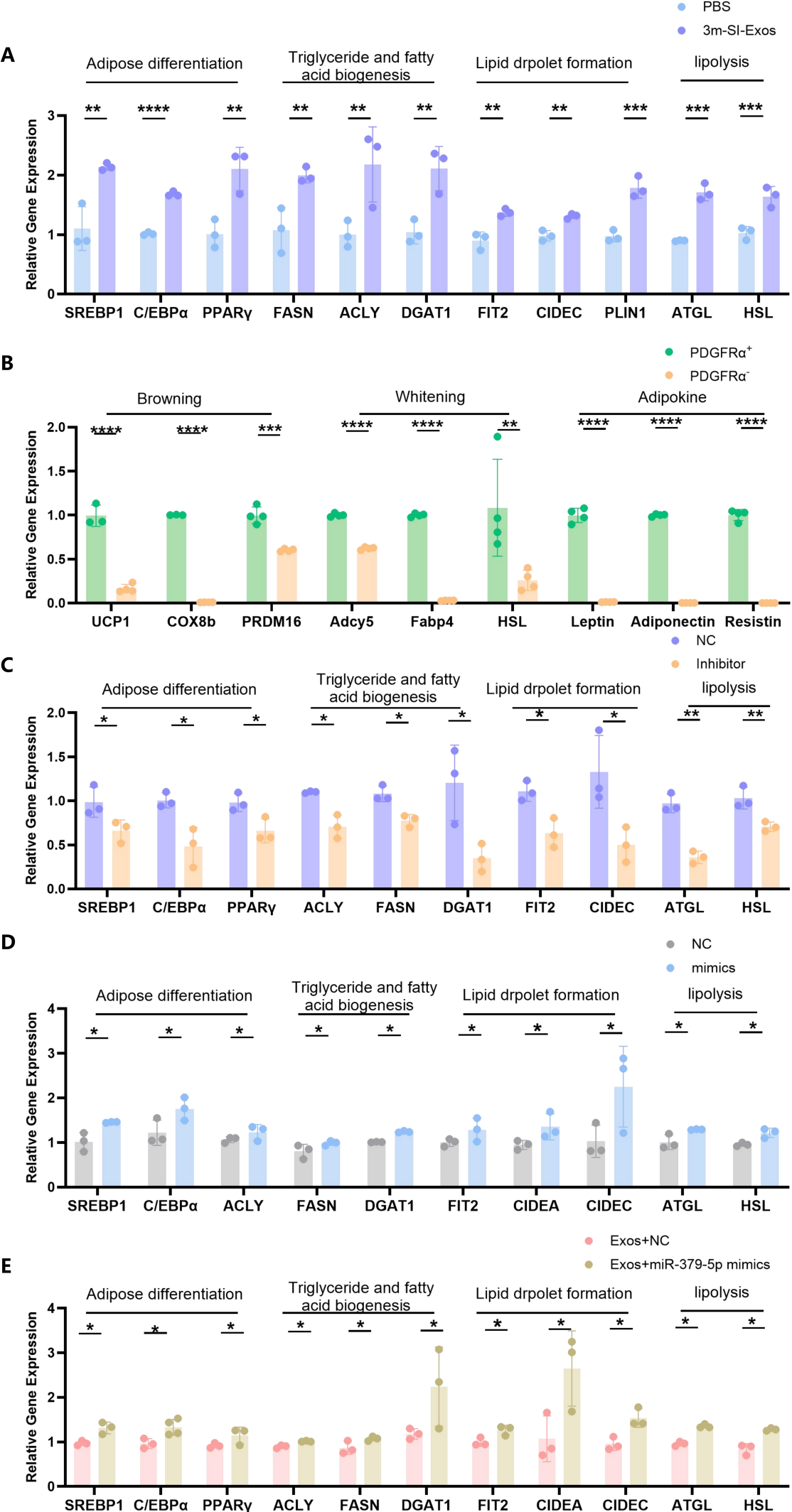


Supplementary Figure 2 Relative gene expressions of lipogenesis related genes. (A) Relative genes expression of adipocyte differential, triglyceride and fatty acid formation, lipid droplet formation, lipolysis in vivo model(n=3). (B) Relative gene expressions of UCP1, COX8b, PRDM16, Adcy5, Fabp4, HSL, Leptin, Adiponectin, Resistin in PDGFRα^+^ progenitor cells and PDGFRα^-^ progenitor cells(n=4). (C)Relative gene expressions of SREBP1, C/EBPα, PPARγ, ACLY, FASN, DGAT1, FIT2, CIDEC, ATGL, HSL in the miR-379-5p interference model(n=3). (D)Gene expressions of SREBP1, C/EBPα, PPARγ, ACLY, FASN, DGAT1, FIT2, CIDEC, ATGL, HSL in the miR-379-5p overexpression model(n=3). (E) Gene expressions of SREBP1, C/EBPα, PPARγ, ACLY, FASN, DGAT1, FIT2, CIDEC, ATGL, HSL in the miR-379-5p overexpression in SI-Exos from aged(20-month-old) mice in PDGFRα^+^ progenitor cells. **p*<0.05, ***p*<0.01, ****p*<0.001.

3m-SI-Exos: small intestinal epithelial exosomes of 3month-old mice. NC: negative control.


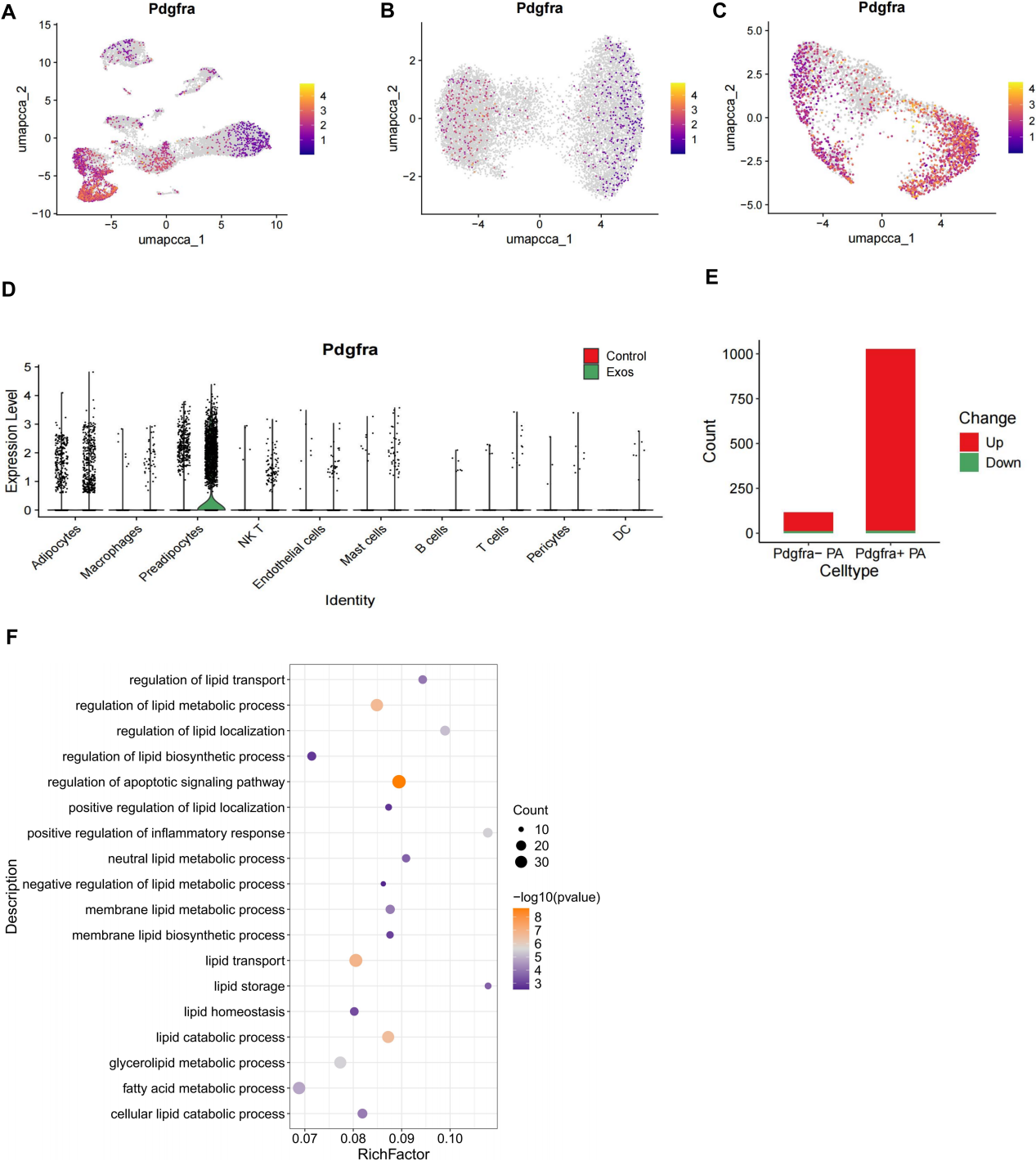


Supplementary Figure 3 scRNA-Seq analysis of SAT, which were isolated from aged mice that had been intravenously injected with PBS or 3m-SI-Exos via the tail vein for 20 injections. (A)Feature plot of PDGFRα in all cells. (B) Feature plot of PDGFRα in adipoctyes. (C) Feature plot of PDGFRα in preadipocytes. (D)Violin plot of expression of PDGFRα in all cells. (E) DEGs of PDGFRα^+^ cell and PDGFRα^-^ cell in preadipocytes. (F) Go term of DEGs of PDGFRα^+^ cell in preadipocytes.

SAT: subcutaneous adipose tissue; 3m-SI-Exos: small intestinal epithelial exosomes of 3months-old mice.


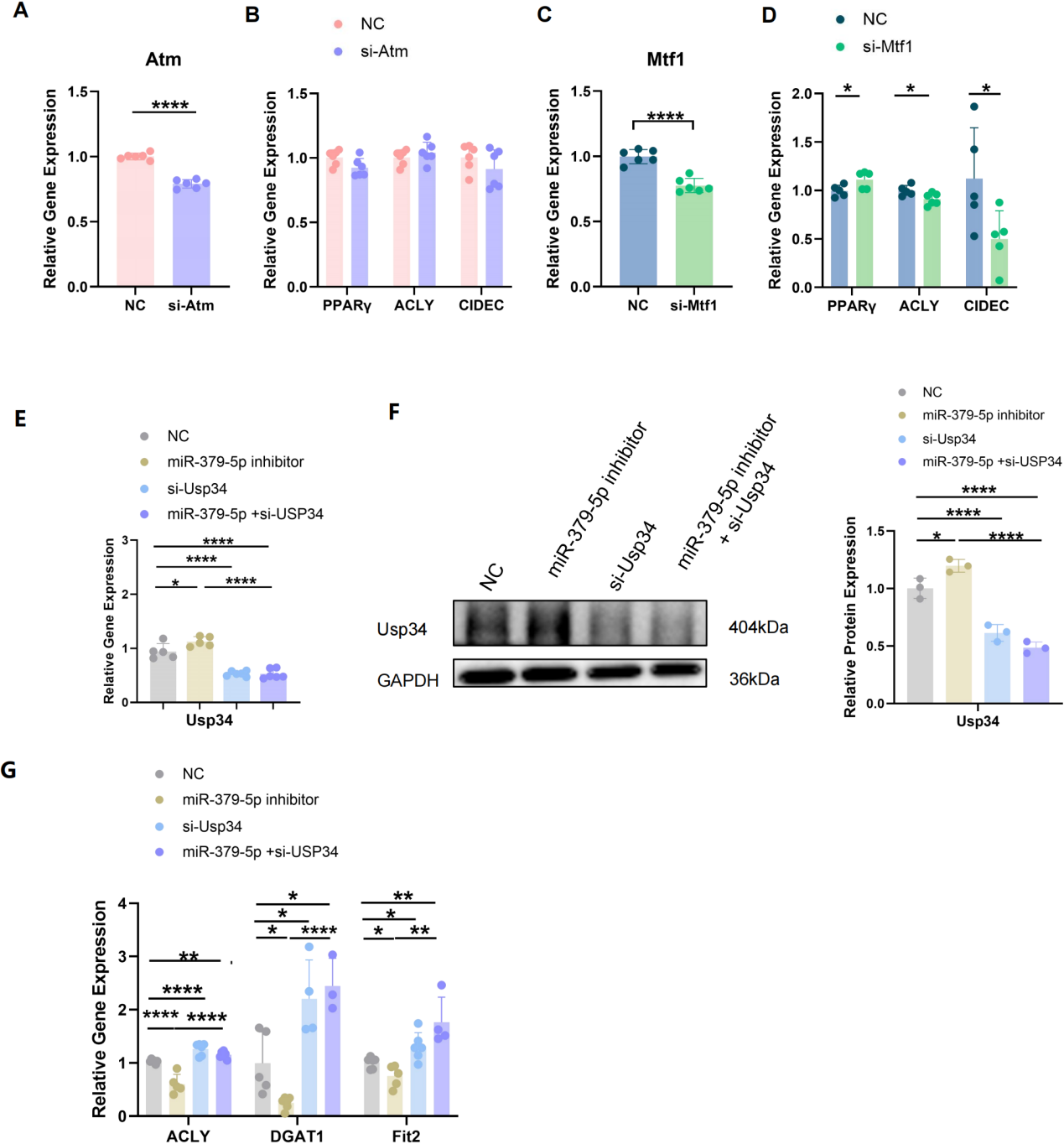


Supplementary Figure 4 MiR-379-5p directly targeted Usp34. (A-B) (Small interfering RNAs (siRNAs) targeting Atm, along with NC, were transfected into PDGFRα^+^ progenitor cells. (A)The intervention efficiency of Atm. (B) Relaitve gene expression level of PPARγ, ACLY, CIDEC. (C-D) siRNAs targeting Mtf1, along with NC, were transfected into PDGFRα^+^ progenitor cells. (C) The intervention efficiency of Mtf1. (D) Relaitve gene expression level of PPARγ, ACLY, CIDEC. (E-G) miR-379-5p, Usp34, and miR-379-5p+Usp34 were individually interfered with in PDGFRα^+^ progenitor cells. (E) The intervention efficiency of Usp34. (F) Relaitve protein expression level of Usp34. (G) Relaitve gene expression level of PPARγ, ACLY, CIDEC. * *p*<0.05，***p*<0.01，****p*<0.001.

NC: negative control; si-Atm: small interfering RNA targeting Atm; si-Mtf1: small interfering RNA targeting Mtf1; si-Usp34: small interfering RNA targeting Usp34.

Supplementary Table 1 Primers used for real-time PCR

| Gene | Prime | |
| --- | --- | --- |
| β-actin | forward | 5’-GGGACCTGTGAGTGCTTCC-3’ |
|  | reverse | 5’-GTATTGAAGAGCCGGGATCTTTT-3’ |
| UCP1 | forward | 5’-AGGGTTTGTGGCTTCTTTTC-3’ |
|  | reverse | 5’-TGGTTGGTTTTATTCGTGGT-3’ |
| Cox8b | forward | 5’-GGAGTGCGACCCCGAGAAT-3’ |
|  | reverse | 5’-CGGCGGAAGTGGGAGTTTT-3’ |
| PRDM16 | forward | 5’-CAGCACGGTGAAGCCATTC-3’ |
|  | reverse | 5’-GCG TGCATCCGCTTGTG-3’ |
| Adcy5 | forward | 5’-CTTGGGGAGAAGCCGATTCC-3’ |
|  | reverse | 5’-ACCGCTTAGTGGAGGGTCT-3’ |
| Adiponectin | forward | 5’-AGGTCTTCTTGGTCATAAGGGTG-3’ |
|  | reverse | 5’-TTGCCAGTGCTGCCGTCATA-3’ |
| HSL | forward | 5’-CCACACGGGAAGAAGACTAGC-3’ |
|  | reverse | 5’-CAGTTGGCCTAGGGTTGGTT-3’ |
| Resistin | forward | 5’-CTTGCCAATCGAGATGACTGT-3’ |
|  | reverse | 5’-GTCTGCCTGAAGCCGTGATAC-3’ |
| PPARγ | forward | 5’-GACCACTCGCATTCCTTT-3’ |
|  | reverse | 5’-CCACAGACTCGGCACTCA-3’ |
| C/EBPα | forward | 5’-GTCACTGGTCAACTCCAGCAC-3’ |
|  | reverse | 5’-CAAGAACAGCAACGAGTACCG-3’ |
| SREBP1 | forward | 5′-TGACCCGGCTATTCCGTGA-3′ |
|  | reverse | 5′-CTGGGCTGAGCAATACAGTTC-3′ |
| PLIN1 | forward | 5’-GGGACCTGTGAGTGCTTCC-3’ |
|  | reverse | 5’-GTATTGAAGAGCCGGGATCTTTT-3’ |
| ACLY | forward | 5’-TTCCTCCTTAATGCCAGCGG-3’ |
|  | reverse | 5’-TGCAGGGATCTTGGACTTGG-3’ |
| FASN | forward | 5’-TGCCTTCGGTTCAGTCTCTT-3’ |
|  | reverse | 5’-CACCCTCCAAGGAGTCTCAC-3’ |
| DGAT1 | forward | 5’-CGTCGCAGAGGTAGTCGTG-3’ |
|  | reverse | 5’-CTACCACCCTGGACGGAAAC-3’ |
| ATGL | forward | 5’-TTCGCAATCTCTACCGCCTC-3’ |
|  | reverse | 5’-AGCAAAGGGTTGGGTTGGTT-3’ |
| FIT2 | forward | 5’-TCCTGCCTTTCATTGCCCTT-3’ |
|  | reverse | 5’-AGTGGCCCGAGATGTCAAAG-3’ |
| CIDEC | forward | 5’-GTGTCCACTTGTGCCGTCT-3’ |
|  | reverse | 5’-TGCTCGCTTGGTTGTCTTG-3’ |
| Klf12 | forward | 5’-CAGCGCCCTTGAGAACAGAAT-3’ |
|  | reverse | 5’-GTGGACGTTTGGAGACCCTTG-3’ |
| C-myc | forward | 5’-GGACCCATCTACAGAGGCTG-3’ |
|  | reverse | 5’-ATCACAATGGTGGAGGGTGC-3’ |
| Gene | Prime | |
| Cyclin D1 | forward | 5’-CATTGTCCCCCGAGATAGCC-3’ |
|  | reverse | 5’-CCATCCTGTCTGGTGGAACC-3’ |
| Fbn2 | forward | 5’-CTCCACCAAAGACGCTCTGG-3’ |
|  | reverse | 5’-CCCTCGTCCCGATACTCAGG-3’ |
| Usp34 | forward | 5’-GATATTGGTGGTCGTTCATGTGT-3’ |
|  | reverse | 5’-TTGGCAAATTCGTAAAGGAAAGC-3’ |
| Atm | forward | 5’-TTGGCAAATTCGTAAAGGAAAGC-3’ |
|  | reverse | 5’-CAGCACACTTCTTTCCACCAC-3’ |
| Mtf1 | forward | 5’-TTTCGTCCTCGAACCAGCTC-3’ |
|  | reverse | 5’-TACACCAGGGAATGCACGTC-3’ |
| Ctnnb1 | forward | 5’-GTTCGCCCCCTTTAATAGTGC-3’ |
|  | reverse | 5’-TGAACTCCAACGTCAAGCGG-3’ |
