# Supplementary material for: From Gut to Fat: Intestinal Epithelial Exosomes Target PDGFRα + Progenitors to Promote Lipogenesis and Counteract Subcutaneous Adipose Tissue Atrophy in Aging
Source: Aging Cell. 2026 Jul 12;25(7):e70625. doi: 10.1111/acel.70625 (PMC13357384; doi:10.1111/acel.70625)
Supplement: Supplementary file 1 — Figure S1: Comprehensive characterization of exosome preparations. (A) Transmission electron microscopy showing typical cup‐shaped morphology with a diameter of approximately 100 nm, consistent with exosome ultrastructure. (B) Nanoparticle tracking analysis revealing a size distribution peak between 100 and 120 nm. (C) Western blot analysis demonstrating enrichment of exosome markers (GPA33, TSG101, and CD63) and the absence of cellular contamination marker (Calnexin). Scale bar, 100 nm. Exos, exosomes. Figure S2: Effects of fecal microbiota transplantation on adipose tissue. Eight‐week‐old germ‐free C57BL/6J mice received FMT from young (3‐month‐old) or aged (20‐month‐old) donors mice, or PBS, twice (200 mg/dose) in 1 week. Subcutaneous adipose tissue was analyzed after one additional week. (A) Representative images and body weights of mice after transplantation. (B) Adipose tissue morphology and weights of SAT and VAT (n = 3). (C) H&E staining of adipocytes from SAT and VAT (n = 3). Scale bar, 50 μm. Error bars represent ± SD. Comparison between two groups was performed by Student's t‐test. *p < 0.05, **p < 0.01, ***p < 0.001. SI‐Exos, small intestinal epithelial exosomes; SAT, subcutaneous adipose tissue; VAT, visceral adipose tissue. Figure S3: Relative gene expression of lipogenesis‐related genes. (A) 20‐month‐old mice received tail‐vein injections of SI‐Exos derived from 3‐month‐old mice (100 μg per injection, every 3 days for 2 months, total 20 injections). Control mice received an equal volume of PBS. Gene expression related to adipocyte differentiation, triglyceride and fatty acid synthesis, lipid droplet formation, and lipolysis (n = 3). (B) PDGFRα+ and PDGFRα− progenitor cells were isolated using magnetic beads and induced to undergo adipogenesis for 6 days. Relative gene expressions of UCP1, COX8b, PRDM16, Adcy5, Fabp4, HSL, Leptin, Adiponectin, and Resistin (n = 4). (C) PDGFRα+ progenitor cells from SAT transfected with miR‐379‐5p inhibitor or negative [file ACEL-25-e70625-s001.zip › Supplementary Table 2.docx]

Supplementary Table 2 Age-related differentially expressed miRNAs

| 102 age-downregulated miRNAs | | | | |
| --- | --- | --- | --- | --- |
| mmu-miR-200b-3p | mmu-miR-1949 | mmu-miR-429-3p | mmu-miR-143-3p | mmu-miR-378a-3p |
| mmu-miR-7b-5p | mmu-miR-20b-5p | mmu-miR-200a-3p | mmu-let-7c-5p | mmu-miR-16-5p |
| mmu-miR-203-3p | mmu-miR-369-5p | mmu-miR-1a-3p | mmu-let-7b-5p | mmu-miR-15b-5p |
| mmu-miR-802-5p | mmu-miR-24-2-5p | mmu-let-7i-5p | mmu-miR-183-5p | mmu-miR-22-3p |
| mmu-miR-203-5p | mmu-miR-467a-5p | mmu-miR-154-5p | mmu-miR-30d-5p | mmu-let-7e-5p |
| mmu-miR-28a-3p | mmu-miR-196a-1-3p | mmu-let-7d-5p | mmu-let-7g-5p | mmu-miR-145a-5p |
| mmu-miR-1983 | mmu-miR-296-3p | mmu-miR-127-3p | mmu-miR-411-5p | mmu-miR-30b-5p |
| novel-mmu-miR380-5p | mmu-miR-802-3p | mmu-miR-139-5p | mmu-miR-26b-5p | mmu-miR-24-3p |
| mmu-miR-378b | mmu-miR-192-3p | novel-mmu-miR220-5p | mmu-miR-10a-5p | novel-mmu-miR433-5p |
| novel-mmu-miR101-5p | mmu-miR-20a-5p | mmu-miR-192-5p | mmu-miR-26a-5p | mmu-miR-150-5p |
| novel-mmu-miR72-3p | mmu-miR-98-5p | mmu-miR-7a-5p | mmu-miR-218-5p | mmu-miR-99b-5p |
| novel-mmu-miR143-3p | mmu-miR-3470a | mmu-miR-9b-3p | mmu-miR-872-5p | mmu-miR-126a-5p |
| mmu-miR-341-3p | mmu-miR-7a-1-3p | mmu-miR-182-5p | mmu-miR-146a-5p | mmu-miR-31-5p |
| mmu-miR-181d-5p | mmu-miR-374b-5p | mmu-miR-375-3p | mmu-miR-194-5p | mmu-miR-195a-5p |
| novel-mmu-miR308-5p | mmu-miR-93-5p | mmu-miR-27b-3p | mmu-miR-191-5p |  |
| novel-mmu-miR75-5p | mmu-miR-379-5p | mmu-let-7a-5p | mmu-miR-1981-5p |  |
| mmu-miR-3068-5p | mmu-miR-25-3p | novel-mmu-miR24-5p | mmu-miR-652-3p |  |
| novel-mmu-miR361-3p | mmu-miR-30c-5p | mmu-miR-3470b | mmu-miR-27a-3p |  |
| mmu-miR-203b-5p | mmu-miR-1839-5p | mmu-miR-191-3p | mmu-miR-196a-5p |  |
| mmu-miR-328-5p | mmu-miR-200c-3p | mmu-let-7d-3p | mmu-miR-23b-3p |  |
| mmu-miR-872-3p | mmu-let-7f-5p | mmu-miR-378c | mmu-miR-34a-5p |  |
| mmu-miR-200b-5p | mmu-miR-151-5p | mmu-miR-200a-5p | mmu-miR-181a-5p |  |

| 30 age-upregulated miRNAs | |
| --- | --- |
| mmu-miR-144-5p | mmu-miR-486b-5p |
| mmu-miR-130b-5p | mmu-miR-214-3p |
| mmu-miR-133b-3p | mmu-miR-144-3p |
| mmu-miR-181a-1-3p | mmu-miR-490-5p |
| mmu-miR-130a-3p | mmu-miR-206-3p |
| mmu-miR-486a-5p | mmu-miR-199a-5p |
| mmu-let-7i-3p | mmu-miR-125b-5p |
| novel-mmu-miR193-5p | mmu-miR-146b-5p |
| novel-mmu-miR118-5p |  |
| novel-mmu-miR53-5p |  |
| novel-mmu-miR142-3p |  |
| novel-mmu-miR328-5p |  |
| mmu-miR-30e-3p |  |
| mmu-miR-671-5p |  |
| novel-mmu-miR112-3p |  |
| mmu-miR-1224-5p |  |
| mmu-miR-1943-5p |  |
| mmu-miR-30e-5p |  |
| mmu-miR-153-5p |  |
| mmu-miR-709 |  |
| mmu-miR-193a-3p |  |
| mmu-miR-141-5p |  |

High-throughput miRNA sequencing was performed on SI-Exos extracted from 3-, 8-, 15-, and 18-month-old mice. SI-Exos, small intestinal epithelial exosomes.
